# Resistance and propulsion performance of a twin skeg ship with different rudder angle

**DOI:** 10.1038/s41598-025-01261-2

**Published:** 2025-06-03

**Authors:** Chen Weimin, Li Yongyue, Xing Lei, Zhang Li, Chang Xing

**Affiliations:** 1 College of Shipbuilding Engineering, Harbin Engineering University, Harbin, 1500011 China; 2 State Key Laboratory of Maritime Technology and Safety, Shanghai Ship and Shipping Research Institute Co., Ltd, Shanghai, China; 3 Shanghai Merchant Ship Design & Research Institute, Shanghai, 201203 China; 4 Key Laboratory of Marine Technology Ministry of Communications, Shanghai, 200135 China

**Keywords:** Computational fluid dynamics, Experimental fluid dynamics, Resistance, Self-propulsion, Hull-propeller-rudder interaction, Twin Skeg, Engineering, Fluid dynamics

## Abstract

Rudder angle will change the flow field, resulting in different loads and interactions on the hull-propeller-rudder, which will affect the resistance and self-propulsion performance of the ship, and this effect will be amplified on the twin skeg ship. Resistance of the ship-rudder at the rudder angle of 0 ° -8 °, lift, moment, axial wake, the rudder profile velocity vector at the propeller shaft height, and the dynamic pressure distribution on the inner and outer surfaces of the rudder were simulated by CFD(Computational Fluid Dynamics) method, and verified by the EFD(Experimental Fluid Dynamics) method. Self-propulsion factors of the hull-propeller-rudder with rudder angle of 0 ° -6 ° were calculated by CFD method, and compared with the EFD results. Axial wake, dynamic pressure on the surface of the propeller and rudder and the velocity vector near the rudder, and the flow field and vorticity field of the hull-propeller-rudder were analyzed. The results showed that : 1 ) The CFD results had the same trend as the EFD results. When the rudder angle was 6 °, the total resistance of the ship-rudder was the smallest, and the resistance decreased by about 1%. 2 ) The change of rudder angle had little effect on the wake field in front, but it had a great influence on the flow field around the rudder, which in turn affected the resistance, lift and moment. 3 ) Self-propulsion performance was the best when the rudder angle was 4 °, and the self-propulsion power can decrease by about 4%, mainly due to the beneficial interaction between the propeller and the rudder. 4 ) Rudder angle had little effect on the surface pressure of the propeller, but it will slightly change the axial wake behind the propeller, and the dynamic pressure of the rudder was quite different. 5 ) When the rudder angle exceeded the optimal rudder angle of 4 °, the interaction between the hull-propeller-rudder became unfavorable, resulting in the chaos of the vorticity field and the overall performance degradation. For twin skeg ships, proper arrangement of rudder angle can effectively improve ship performance and achieve energy saving purpose.

## Introduction

With the strict requirment of EEDI regulation, merchant ships should have good performance to reduce the energy consumption, therefore, ships with low carbon have been very popular. Energy-saving devices are more common in single-propeller merchant ships, and rarely installed on twin-propeller ships due to layout limitations, but energy conservation and emission reduction of twin-propeller ships are also important. Compared with single-propeller ships, twin-propeller ships have many advantages in ship maneuverability, propulsion efficiency and safety. For instance, due to the limitation of the propeller diameter for ships in restricted waterways, the twin-propeller ship form can effectively improve the propulsion efficiency. This applies to large-capacity shallow draft ships transporting light cargoes, as well as some full large engineering ships. The main function of the propeller is to provide propulsion thrust for the ship, which determines the energy consumption of the ship. The rudder is mainly used for keeping course and maneuvering of ships, especially for twin-propeller ships^1^. The rudder type has effect on maneuverability and seakeeping performance^2,3^. The layout of the rudder also has an impact on the ship speed performance, which results from the impact of the rudder on the ship resistance and the rectification of the rudder on the propulsion efficiency^2,4–7^. It is known from the model test that the influence of the rudder on the resistance performance of single-propeller ship is roughly 1 -2%. For ships with twin propellers and twin rudder, the rudders at the symmetrical positions on the port and starboard also have an important influence on the resistance performance and self-propulsion performance of the ship. The contribution of the streamlined rudder to the hull resistance is approximately 2 -4%. When the combined hull-rudder and hull-propeller-rudder system is considered, the change of the flow field is more complicated. In the ship design stage, the hull-propeller-rudder may cooperate and interfere with each other due to different design types, and thus the overall performance of the ship is affected by the hull-propeller-rudder system. When the twin rudder is arranged, the flow field near the rudder changes, which in turn affects on the rudder itself, the hull and the propeller, i.e. hull-propeller-rudder interaction^8–11^.

Both CFD and EFD are good methods for ship performance analysis and flow field analysis. Molland and Turnock shows that the lift coefficient of the open-water rudder and the rudder behind the propeller are approximately linear to the attack angle, while the drag coefficient roughly has an parabolic relationship with the attack angle. In addition, the propeller has an effect on the position of the pressure center of the rudder^12^. Park and Chun improved the propulsive performance of twin-skeg hull form by deriving the hydrodynamic standards, and studyed the influence of stern arrangement forms to the propulsion efficiency^13^.Charles et al. used CFD method to simulate different inflow angles of single-propeller and single-rudder system. Various angles between the propeller and the rudder have also been investigated. The change of the rudder angle can obviously alter the lift coefficient and drag coefficient of the rudder, as well as the thrust coefficient of the propeller^14^. The research by Guo and Li demonstratess that numerical simulation is a good method to study ship resistance and self-propulsion^15,16^. Jialun and Robert ‘s research reveals that due to the rotation of the propeller, the inflow attack angle on the rudder surface changes. As a result, there should be an optimal arrangement angle between the rudder and the propeller to obtain the minimum resistance coefficient, which can further reduce the total resistance of the ship^2^. Jialun ‘s research on single-propeller twin-rudder ships presents that there is a maximum utility range for the attack angle of the rudder. In the range of ± 15 °, the lift coefficient increases with the increase of the attack angle, while the drag coefficient does not change much, which means that there is an optimal arrangement of the rudder angle^17^. Yue Jiao designed and developed the rudder system. The numerical results demonstrate that the fluid behind the propeller generates torque on the rudder. The lift performance of the rudder is affected by the wake and working state, which has different degrees of beneficial effects on the propulsion performance of the ship^18^. Wu Sichuan studied the influence of the rudder angle of the double podded propulsor on the self-propulsion performance of medium-sized luxury cruise ships. It is shown that an optimal rudder angle exists between the power received by the ship and the podded propulsor^19^. Nobuaki Sakamoto et al. used the overset RANS to simulate and validate the hull-rudder interaction coefficients, the results show that the RANS method has acceptable accuracy for simulating hydrodynamic coefficients^20^. Sadakata, Hino and Takagi used the CFD method to evaluate the the energy-saving devices efficiency, the results showed that CFD method is capable of predicting the performance of ship simulations^21^.

Vortex systems and wake field of propeller-rudder interaction was investigated by CFD method, which can help to give the details of interaction phenomena of the flow field, and to predict the unsteady loads^22^. Roberto et al. used the CFD method to invetigate the propeller-rudder interference phenomenon of the twin-propeller ship. It is indicated that the co-directional rudder angle between the two rudders has a great influence on the force of the rudder and has obvious interference with the wake field^23^. Diego et al. investigated the interference of open water rudder and the propeller-rudder based on the RANS approach. The larger rudder angle has a significant influence on the performance of the open water rudder and the rudder behind the propeller^24^. Hu Jian et al. analysied the pressure distribution and wake flow field of single propeller-rudder interaction under different angles. The results show that, rudder angle mainly affectted the resistance and lift force of the rudder, had minor influence on propeller propulsion characteristics. When the rudder angle was about 5 degree, the rudder resistance was smallest^25^. The research of Naz Yilmaz et al. investigated propeller-rudder-hull interaction by using CFD and EFD methods, including the propeller performance in cavitation conditions. The results show that CFD method and EFD method can play a important role in cavitation observations^26^. In the oblique flows, the hydrodynamic coefficients of propeller and rudder would incerease under flow angles, the vortex street would shift as the angle changed, but not completely coincide with the flow angle. Therefore, flow angle on the propeller or rudder changed the performance of propeller or rudder^25^. The propeller-hull interaction was described and compared for three different propulsion system of a twin-propeller/ twin-rudder ship, based on CFD approach. The propeller worked in the wake generated by the hull shape, it’s propulsive factors had difference with it’s open water characterstics^27^. Andrea Franceschi et al. investigated the main interaction effect of hull, propeller and rudder. The flow angle not only had impact on the manoeuvring performance, but also affected the performance and velocity distribution of the rudder^28^. Zhiqiang Liu et al. optimized the hull form resistance and wake distortion by considering hull-propeller interaction, in which, the pressure distribution of propeller and the vortex form changed with the hull form transformed, leading to the optimization of self-propulsion performance^29^. To study the propeller-rudder interaction under different rudder angles, Hu Jian et al. simulated the propeller-rudder cases of different rudder angles. The results demonstrated that larger rudder angle results in higher hydrodynamic performance of the propeller and higher resistance coefficient of rudder^30^. Sakamoto et al. estimated the Low L/B Twin-Skeg Container Ship by using RANS method, proving the RANS method accurately predicted the resistance and self-propulsion coefficients and the flow field^31^. And also, CFD method can be a practical and powerful tool for merchant ship numerical simulations^32^. The above research indicatess that the rudder angle has a direct impact on the rudder, propeller and wake field, which provides a research basis for the present study. For twin-propeller / twin-rudder ships, the change of rudder angle will have a significant impact on the interaction of hull-propeller-rudder, and have a direct impact on the resistance and self-propulsion performance of the ship, thus affecting the energy consumption of the ship. This impact of the rudder angle, under the symmetrical arrangement, on the resistance, self-propulsion performance and hull-propeller-rudder interaction, is relatively less and deserves more research.

To analyze the resistance and self-propulsion performance and the hull-propeller-rudder interaction of the twin-propeller/twin-rudder ship under different rudder angles, the CFD and EFD analysis were carried out at model scale, ITTC(International Towing Tank Conference) recommended procedures were referred in the CFD simulations and EFD model tests. For the resistance characteristics and flow field of hull-rudder, the hull-rudder resistance, axial wake field, velocity vector around rudder and dynamic pressure distribution on rudder surface at different rudder angles were studied, and the resistance results of CFD and EFD are compared. Propeller open water characteristics and self-propulsion factors, rotational speed, thrust and torque of hull-propeller-rudder force balance at different rudder angles were studied and compared. The axial wake distribution in front of the rudder, the dynamic pressure distribution of the propeller surface and rudder surface, and the velocity vector around the rudder were analyzed. In order to further analyze the interaction between hull-propeller-rudder, the streamline and wake vortex around hull-propeller-rudder at different rudder angles were obtained. At last, the present study can provide insights for energy saving and emission reduction of ship design.

## Methods and theories

### Main diementions of the ship, rudder and propeller

**Fig. 1:**
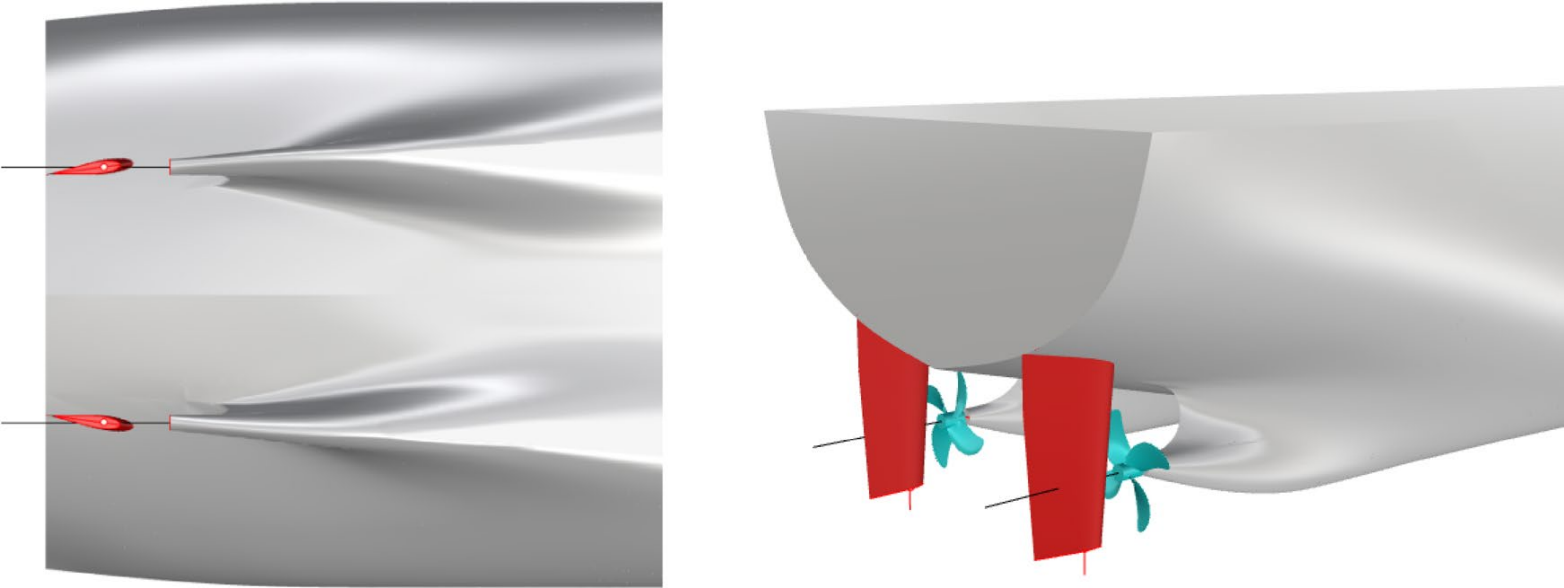
Arrangement of the ship, propellers and rudders. Source: Fig. 1 developed by the authors. (**a**) Hull with rudders (**b**) Hull with propellers and rudders.

The main parameters of the ship model are shown in Table 1. The general layout of the stern part is demonstrated in Figure 1. Owing to the symmetry of the twin-propeller ship, the rudder angle is positive counterclockwise viewed from the port rudder and the starboard rudder rotates symmetrically.

**Table 1:** Main parameters of the ship model.

Data	Value	Data	Value
Model Length (m)	6.900	Breadth (m)	1.072
Draft (m)	0.269	Block Coefficient	0.753
Speed (m/s)	1.534	Froude number	0.187
Reynolds number	1.084 ×10^7^	Propeller Diameter(m)	0.197
Number of Propellers	2	Propeller Blades	4
Propeller Rotation	Inward	Pitch Ratio at 0.7R	0.917
Propeller Chord at 0.7R(m)	0.0486	Propeller Thickness(m)	0.0026
Rudder Profile	NACA	Rudder Angle(°)	0, 2, 4, 6, 8

### Analysis framework

**Fig. 2:**
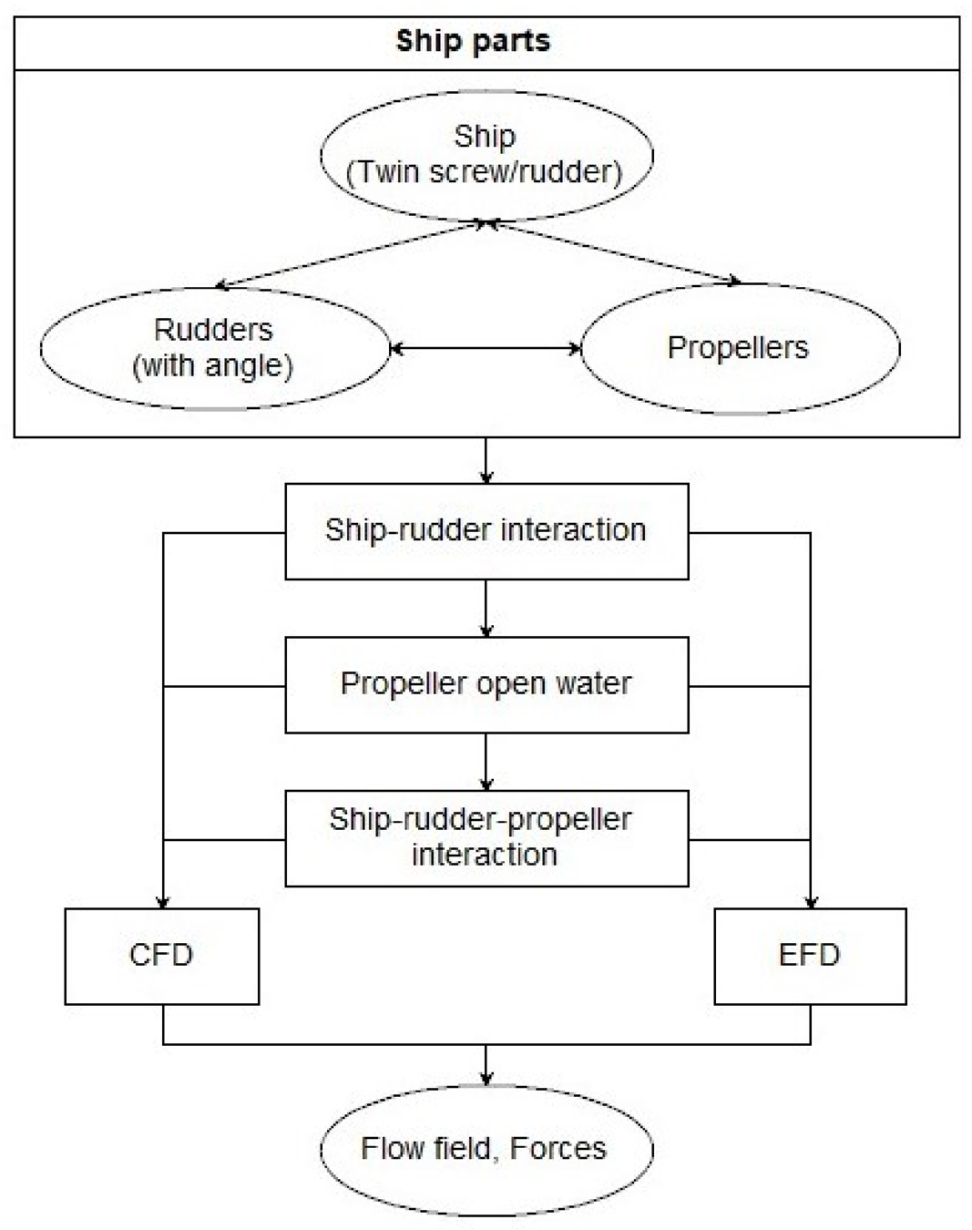
Flow chat of analysis modular approach.

Hull-propeller-rudder interaction is a complex hydrodynamic problem, which affects the overall performance of the ship. In order to study the influence of the rudder angle impact on the hull-propeller-rudder interaction, and analyze the influence on the overall performance of the ship at the same time, a modular analysis framework is carried out, as shown in Fig. 2:

1. The input components are hull, twin propellers and twin rudders.
2. The change factor is symmetrical rudder angle.
3. When hull-rudder is integrated, CFD method and EFD method are used to analyze the resistance, lift, flow field and other information of hull and rudders.
4. When the ship-propellers-rudders are installed, propeller open water characteristics and self-propulsion performance of the ship are calculated by CFD method and EFD method, and the force and flow field of different components are analyzed, self-propulsion factors are calculated and compared.

### CFD theories

#### Turbulence model

As a widely used turbulence model in industry, Reynolds-Averaged Navier-Stokes (RANS) method averages the turbulence scale, which reduces the computational difficulty and enhances its capability to solve complex ship hydrodynamic problems. RANS method is recommended by ITTC for the numerical calculation of ship resistance and flow field^33,34^. Among the different types of RANS methods, the SST k-$\:\omega\:$ model is more common because of its good adaptability to near-field and far-field solutions. The governing equations are as follows :

1 $$\frac{{\partial \rho }}{{\partial t}}+\nabla \,\,\left( {\rho \overrightarrow v } \right)=0$$

2 $$\frac{\partial }{{\partial t}}\left( {\rho \overrightarrow {\text{v}} } \right)+\nabla \,\,\left( {\rho \overrightarrow {\text{v}} \otimes \overrightarrow {\text{v}} } \right)= - \nabla \,\overline {{{p_{\bmod }}}} \overrightarrow {\text{I}} +\nabla \,\,\left( {\overrightarrow {\text{T}} +{{\text{T}}_{RANS}}} \right)+{\overrightarrow f _b}$$

In the above formulae, *ρ* $\nabla$is the density, is the Hamiltonian operator, $\:\stackrel{-}{V}$ is the average velocity, $\:{p}_{\text{m}\text{o}\text{d}}$ is the modified pressure, *I* is the unit tensor, $\:\stackrel{-}{T}$ is the average viscosity tensor, *T*_RANS_ is the additional stress term, and *f*_b_ is the object force, such as gravity. Implicit unsteady solver is used for modeling time, and finite volume method is used for space discretized.

#### Free surface simulation

The volume of fluid (VOF) model is a method to solve the problem where a ship encounters a gas-liquid two-phase flow. The VOF model was employed in both resistance and self-propulsion simulations. By capturing the two-phase flow, the distribution and movement of the immiscible interface can be predicted^35^. Assuming that the volume fraction of one phase i is described by the field of α_i_, the volume fraction is,

3 $${\alpha _i}=\frac{{{V_i}}}{V}$$

Where *V*_i_ is the volume of the grid cell occupied by phase *i*, and *V* is the total volume of the cell.

The total mass conservation equation of all phases is given as:

4 $$\frac{\partial }{{\partial t}}\left( {\int\limits_{V} {\rho dV} } \right)+\oint\limits_{A} {\rho \overrightarrow {\text{v}} } \,d\overrightarrow a =\int\limits_{V} {SdV}$$

5 $$S=\sum\limits_{i} {{S_{\alpha i}}\,{\rho _i}}$$

Where, $\:\overrightarrow{v}$ is the fluid velocity vector, $\:\overrightarrow{a}$ is the surface area vector and *S* is the mass source term.

6 $$\begin{gathered} \frac{\partial }{{\partial t}}\left( {\int\limits_{V} {\rho dV} } \right)+\oint\limits_{A} {\rho \overrightarrow {\text{v}} } \otimes \overrightarrow {\text{v}} \,d\overrightarrow a = \hfill \\ - \oint\limits_{A} {p\overrightarrow {\text{I}} } \,d\overrightarrow a +\oint\limits_{A} {\overrightarrow {\text{T}} } \,d\overrightarrow a +\int\limits_{V} {\rho gdV} +\int\limits_{V} {{{\text{f}}_b}dV - \sum\limits_{i} {\int\limits_{A} {{\alpha _i}{\rho _i}} } } \overrightarrow {{{\text{v}}_{d,i}}} \otimes \overrightarrow {{{\text{v}}_{d,i}}} \,d\overrightarrow a +\int\limits_{V} {S_{i}^{\alpha }dV} \hfill \\ \end{gathered}$$

Where, *p* is the pressure, *I* is the unit tensor, *T* is the stress tensor, *f*_b_ is the vector of the body force and $\:{S}_{i}^{\alpha\:}$ is the phase momentum source term.

#### Simulation of the rotating propeller

**Fig. 3:**
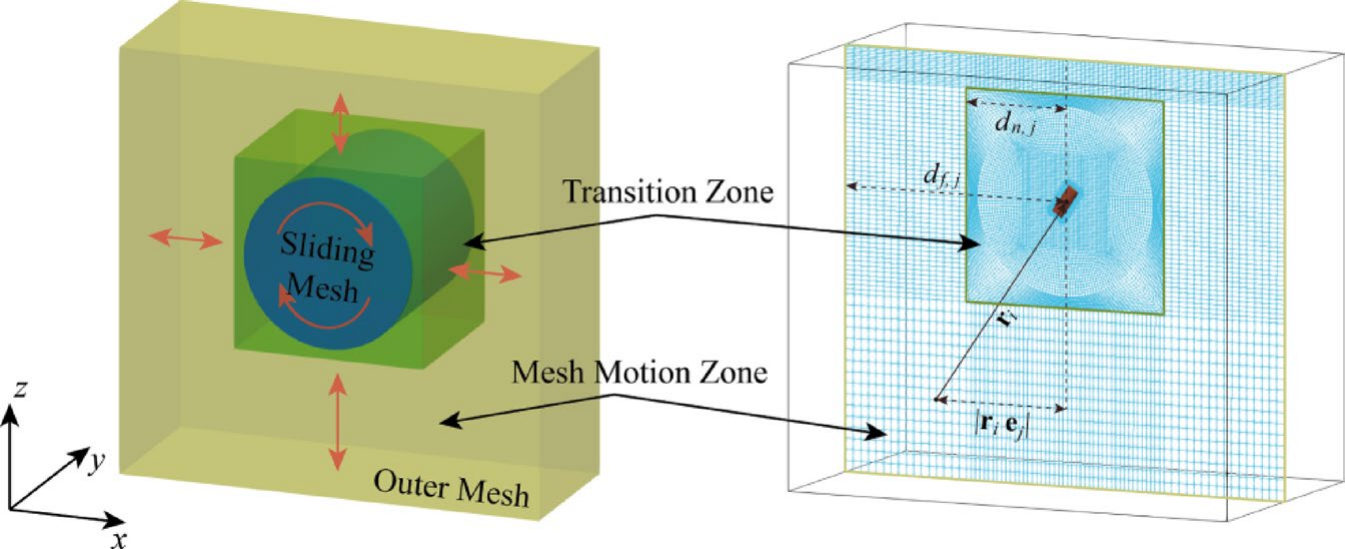
Sliding mesh illustration of RBM method. Source: Fig. 3 illustrated by Fan et al.^36^.

In order to simulate the propeller propulsion and flow field changes in the self-propulsion state more realistically and improve the computational accuracy, the Rigid Body Motion ( RBM ) method is used to simulate the rotation of the propeller behind the ship^36^. This approach can capture the propulsion characteristics of the propeller in the non-uniform flow field. The propeller is considered as a rigid body, and the rotating domain wrapping the propeller is defined outside the propeller. The rotating motion of different speeds is imposed to monitor the thrust and torque generated by the propeller at different speeds. The RBM method uses sliding mesh method. There is a sliding interface around the propeller rotation domain, and data transmission occurs on the interface. The mesh area around the propeller rotates in real time, and the mesh rotation speed is the propeller rotation speed, which can capture the transient flow field. Therefore, data exchange occurs without the movement of grids in different regions. The illustration of Fig. 3 shows the theory of RBM method.

### Experimential theories

**Fig. 4:**
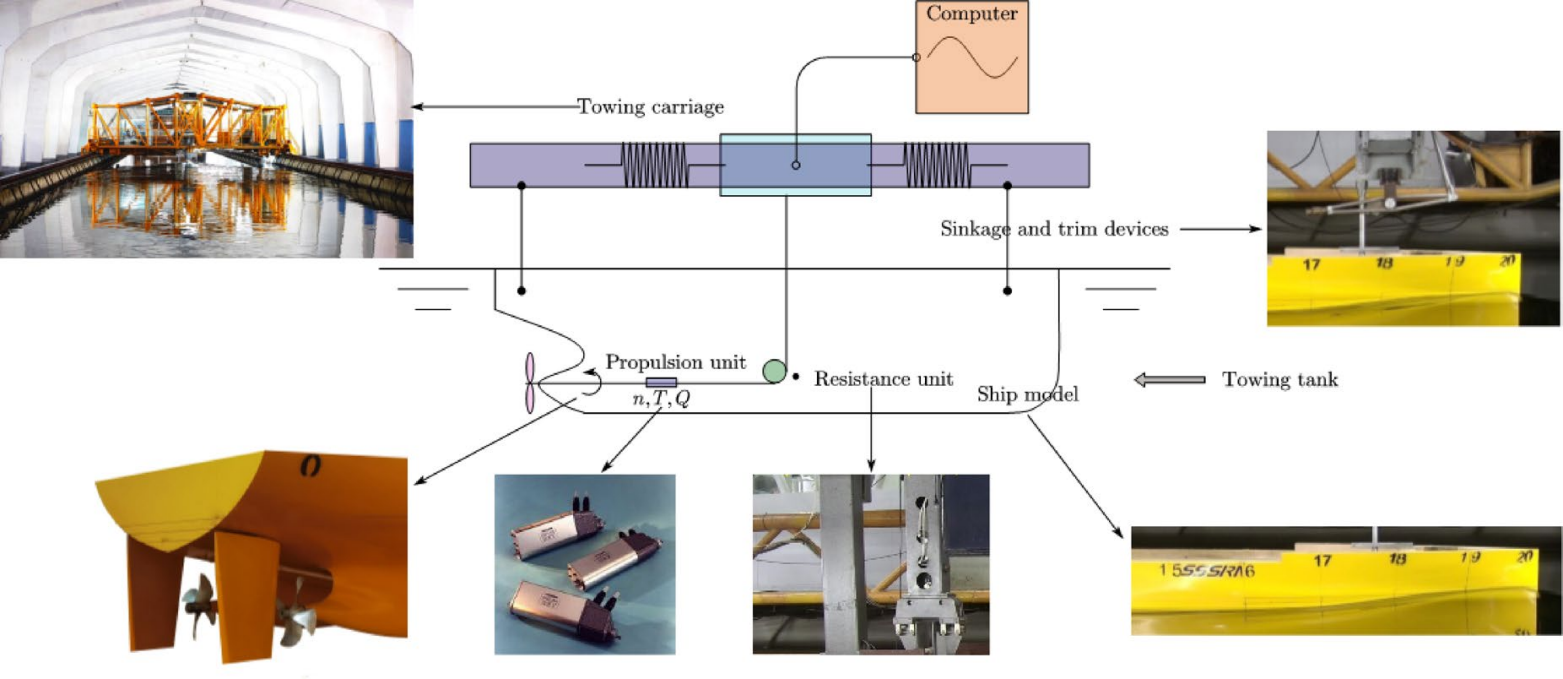
Facilities of the resistance tests and self propulsion tests. Source: Fig. 4 illustrated by the authors.

The model test method is often used as the benchmark line. It can effectively verify the results of the design scheme. The model test facilities include towing tank, resistance dynameter and self-propulsion instrument. The model test process is based on the ITTC recommended procedures. The facilities of resistance and self-propulsion tests are shown in Fig. 4. The resistance reference procedure is *Resistance tests*^37^, the version is 2021, and the procedure number is 7.5-02-02-01. For the ship model resistance test, the rudders were installed on the ship model, and the ship model was connected to the towing carriage and ballasted in water. At the corresponding speed, the total resistance at different rudder angles is measured by the resistance dynameter unit. The self-propulsion tests reference procedure is *Propulsion/Bollard Pull Test*^38^, the version is 2021, and the procedure number is 7.5-02-03-01.1. For the self-propulsion tests, the propellers were installed on the ship model, and the self-propulsion unit was installed behind the ship model to measure the rotational speed, thrust and torque of the ship model at the corresponding speed.

**Fig. 5:**
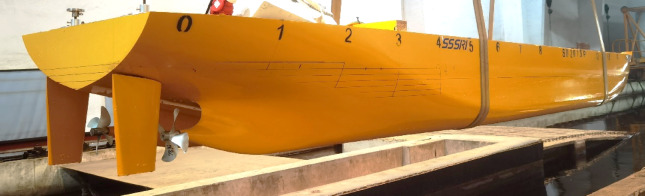
Ship model tests. Source: Fig. 5 taken by the authors.

**Fig. 6:**
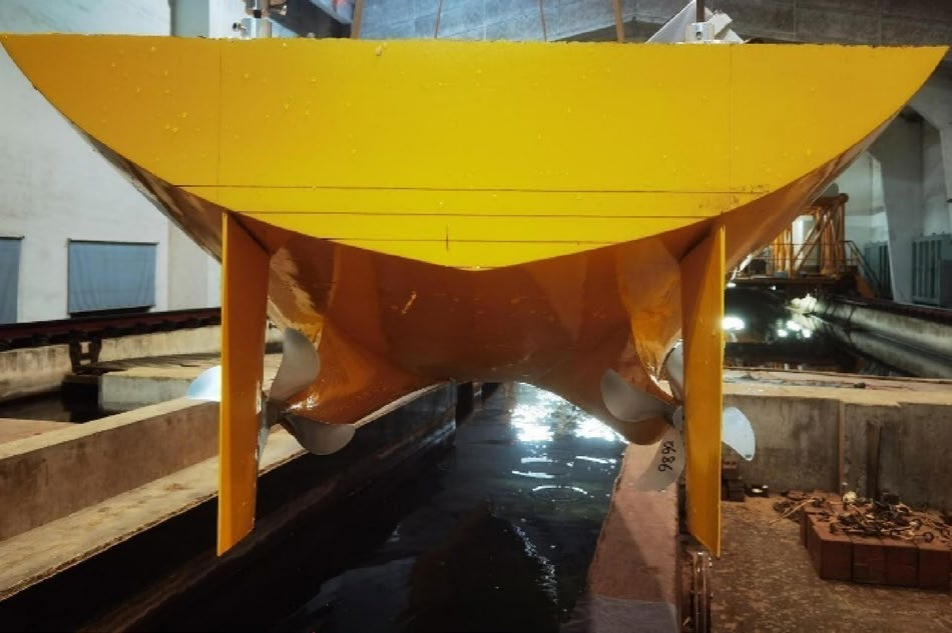
Aft body of the ship model. Source: Fig. 6 taken by the authors.

The model tests of the twin propeller/twin rudder ship model is shown in Figs. 5 and 6. The model tests were conducted in Shanghai Ship and Shipping Research Institute Co., Ltd. The main dimensions of the towing tank are : length 192 m, width 10 m, water depth 4.2 m. The speed range of the carriage is 0–9 m / s, the resistance dynamometer range is 0–200 N, The open-water dynamometer has a rotation range of 0-3000 rpm with maximum thrust 400 N and maximum torque 15 N·m. The self-propulsion measurement range of rotation is 0-3000 rpm, the thrust range is 0–250 N, the torque range is 0–10 N·m.

## Impact of Rudder angle on resistance performance and hull-rudder interaction

### Diagram of the hull-propeller-rudder

**Fig. 7:**
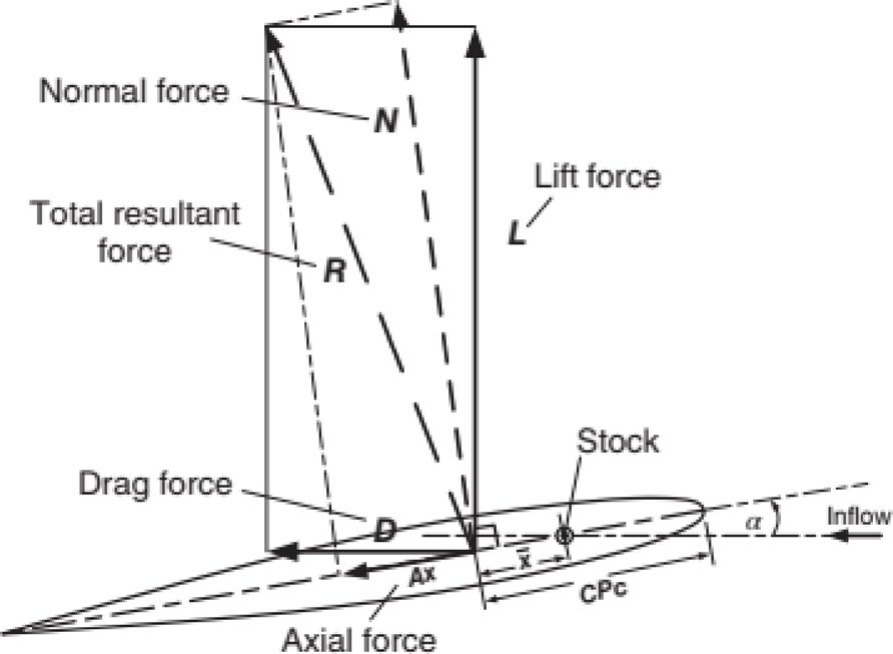
Schematic diagram of rudder force. Source: Fig. 7 cited from Molland and Turnock^12^.

**Fig. 8:**
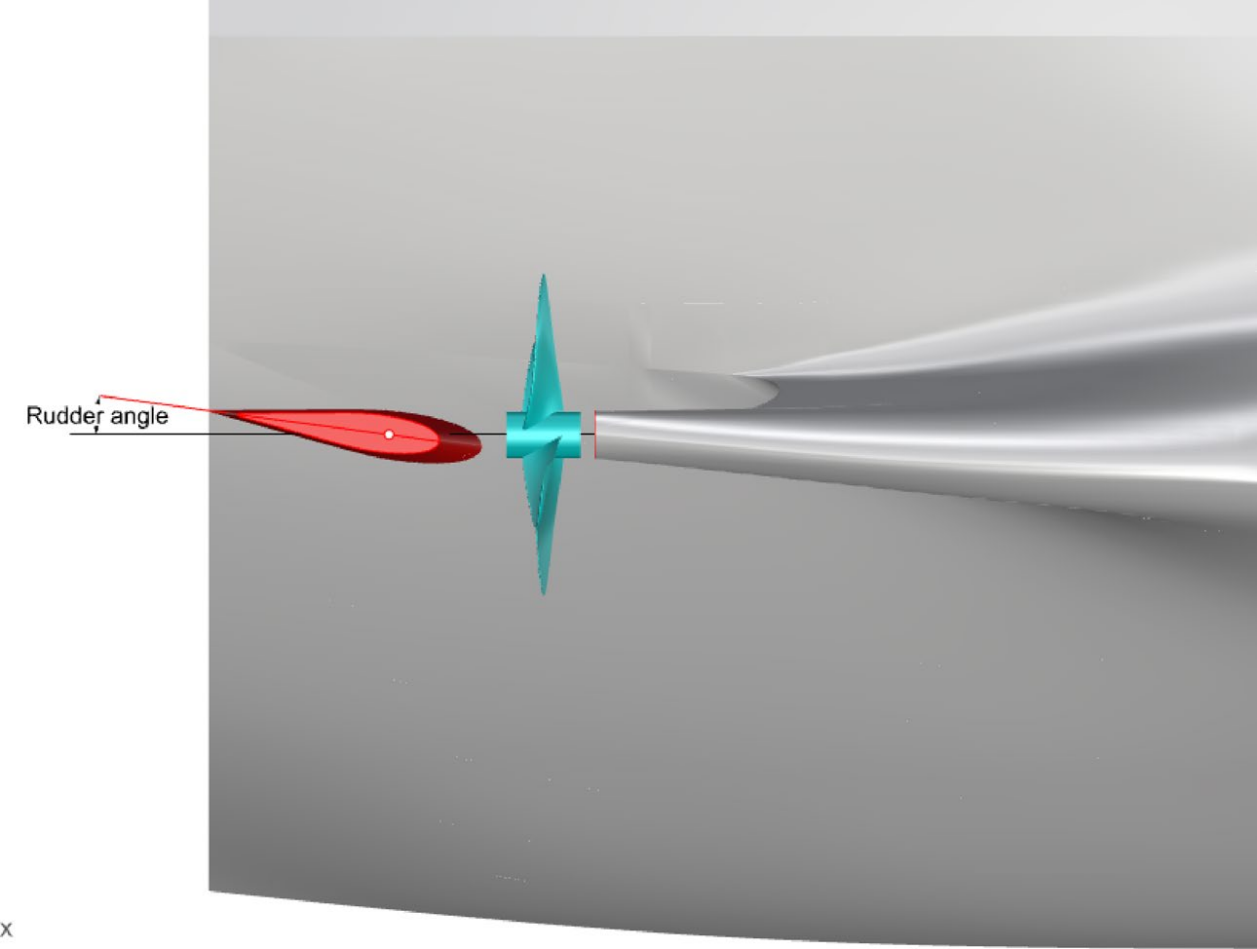
Port of hull-propeller-rudder. Source: Fig. 8 drawn by the authors.

As shown in Figs. 7 and 8, α is the angle between the rudder centerline and the incoming flow direction, which is positive counterclockwise. Stock givess the rudder stock position. *D* and *L* represents drag and lift forces along the inflow direction, respectively. *N* is the normal force perpendicular to the center line of the rudder section. *A*_x_ is the axial force along the center line of the rudder section and *R* is the total force of the rudder.

### Numerical simulations and experimental tests of resistance performance

**Fig. 9:**
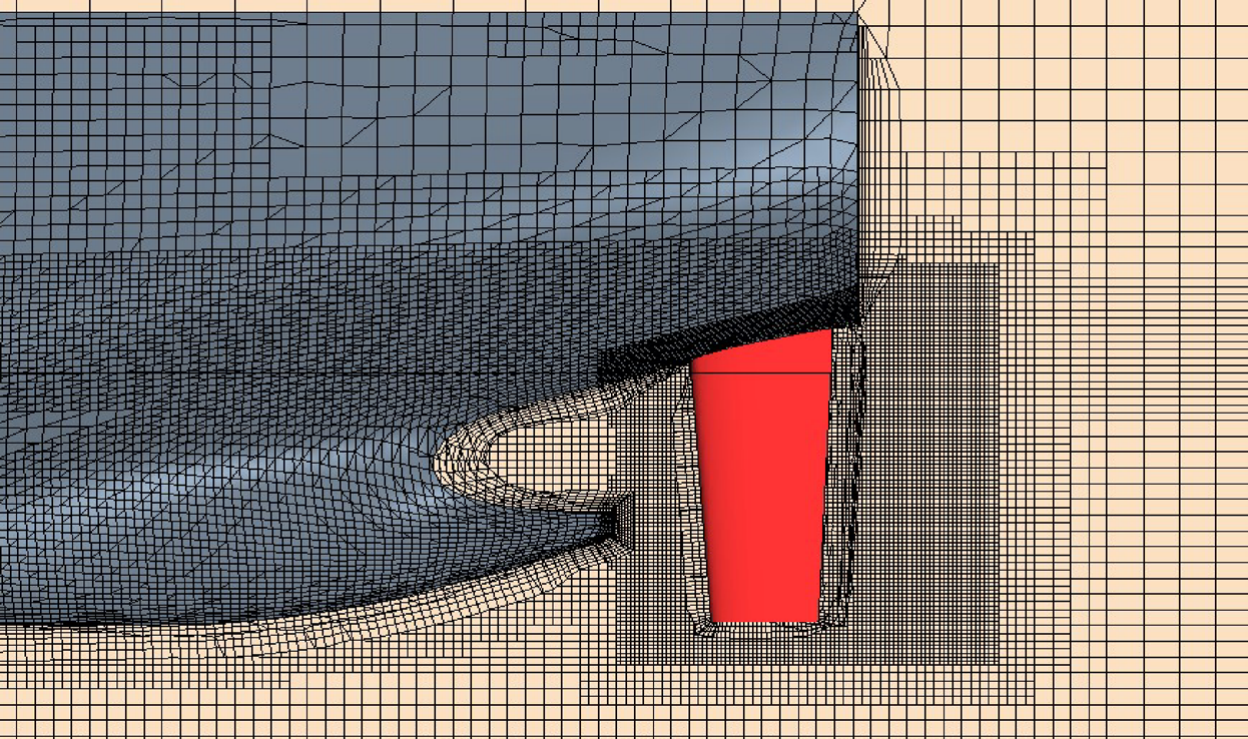
Mesh of the aft body of the ship.

**Fig. 10:**
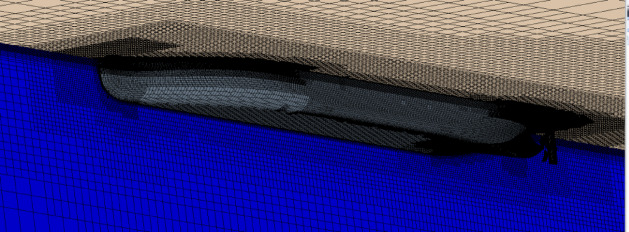
Mesh of the overall computational domain.

Firstly, the resistance characteristics of the model are analyzed. Based on the star-ccm+, the SST k-ω turbulence model is used to calculate the flow field. The mesh near the free surface, the hull and the rudder area are refined, as shown in Figs. 9 and 10. The inflow face, domain side and domain bottom were set as the inlet velocity conditions, the outflow was the pressure outlet condition, the ship and rudder surface were set as the wall condition. The domain captured the half hull in the middle plane along the ship. The upstream domain was 1.4×Lpp, the downstream domain was 2.5×Lpp, side domain about 1.5×Lpp. The mesh zone near the ship, rudder, wake and free surface were refined. The near wall had 8 prism layers and the minimum wall spacing was about 7.2e-5 m. When calculating the self-propulsion simulations, the zone around the propeller was set as the rotating zone, the rotating speed was same as the propeller rotation speed. The density was 998.00 kg/m^3^, the dynamic viscosity was 9.78E-4 Pa·s.

**Fig. 11:**
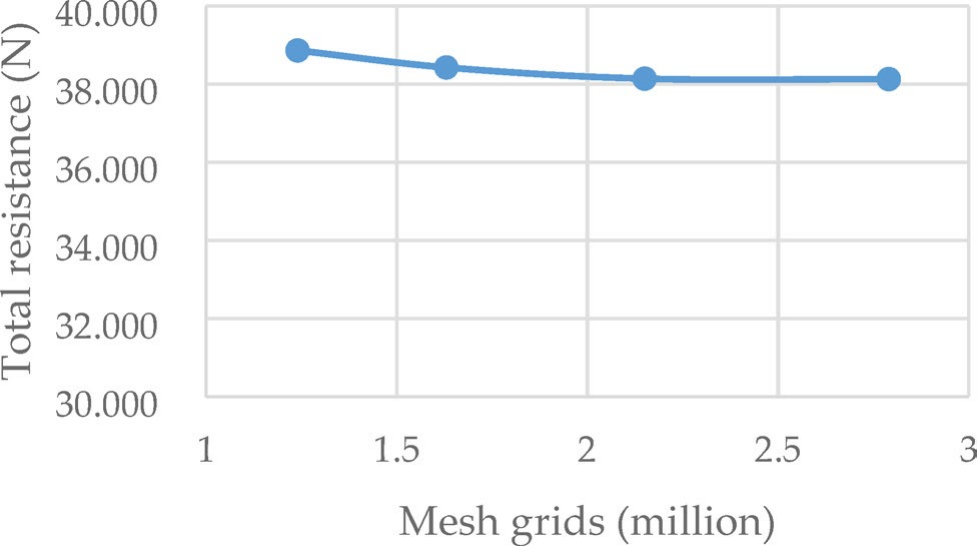
Numerical results of different case with different grid density.

The numerical domain is meshed for half hull. The grid convergence study is performed at the rudder angle 0°. For the convergence verification of the numerical calculation grid, refer to the verification and validation procedure in the ITTC procedures^33,34,39^, the calculation steps are as follows. The verification and validation procedure is 7.5-03-01-01, namely *Uncertainty Analysis in CFD*,* Verification and Validation Methodology and Procedures*^33^. For the convergence studies, four sets of grids are generated, respectively, they are coarse, medium and fine grids. *R*_G_ is defined as the convergence ratio, as shown in Eq. (7). With the increase of the number of grids, the total resistance shows a monotonic convergence trend, as shown in Table 2; Fig. 11. In order to reduce the calculation workload, the grid number of 2.15 million is selected for the following resistance calculation.

7 $${R_G}=\frac{{{\varepsilon _{21}}}}{{{\varepsilon _{32}}}}=\frac{{{S_{GM}} - {S_{GF}}}}{{{S_{GC}} - {S_{GM}}}}$$

Where, *S*_*GF*_, *S*_*GM*_, *S*_*GC*_ are the solutions of the fine grid, medium grid, and the coarse grid. When 0 < *R*_*G*_<1, the results were monotonic convergence. If *R*_*G*_ <0, the numerical results oscillatory convergence. If *R*_*G*_ >1, the numerical results were divergent.

**Fig. 12:**
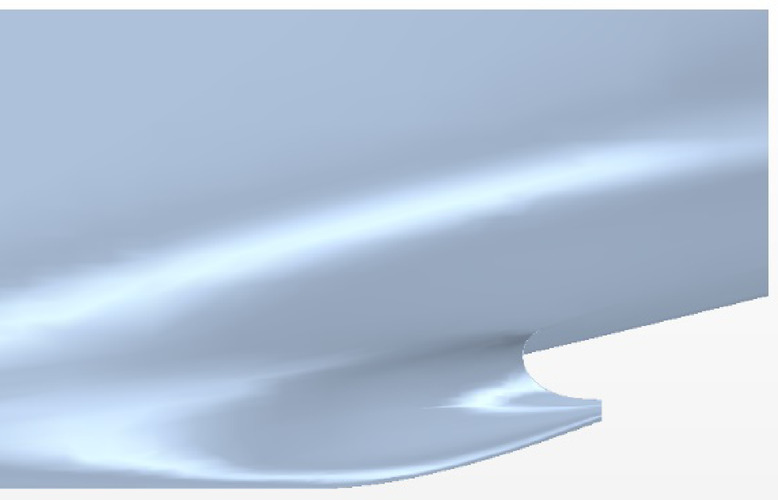
Arrangement of ship aft body (bare hull).

**Fig. 13:**
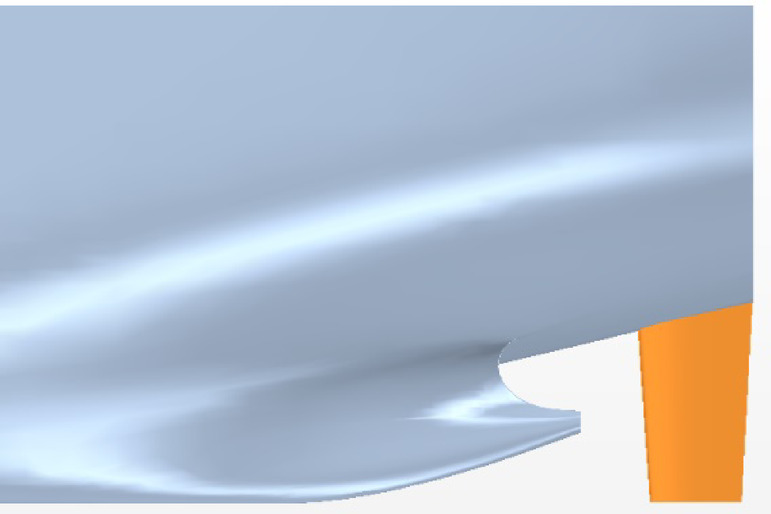
Arrangement of ship aft body and rudder.

In order to compare the resistance performance with different arrangements, the numerical simulation of bare hull and hull-rudder schemes with rudder angle of 0°, 2°, 4°, 6°, 8°, were carried out, as shown in Figs. 12 and 13. The total re-sistance, rudder surface pressure, rudder profile velocity vector and other information were analyzed. 

**Table 2:** Grid convergence validation of the calculation domain.

Grids (million)	Total resistance (*N*)	*R*_G_
1.24	38.861	–
1.63	38.426	–
2.15	38.139	0.660
2.79	38.128	0.038

**Fig. 14:**
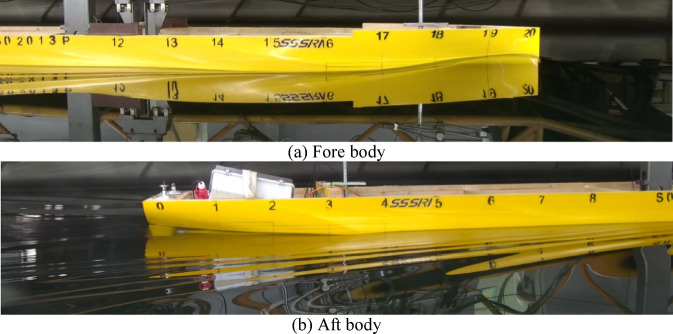
Ship model tests. Source: Figure 14 taken by the authors.

### CFD and EFD results of different Rudder angle cases

The total resistance results of the bare hull and the scheme with different rudder angles are shown in Table 3.

**Table 3:** Results of different Rudder angle case.

Data	Rudder angle					
	Bare hull	0°	2°	4°	6°	8°
Total resistance -EFD(N)	–	37.719	37.612	37.414	37.366	37.497
Total resistance -CFD(N)	36.301	38.139	38.081	37.798	37.725	37.903
Rudder resistance -CFD(N)	–	1.838	1.780	1.497	1.424	1.602
Rudder lift -CFD(N)	–	3.934	3.925	1.465	1.000	3.300
Rudder moment -CFD(N*M)	–	0.0656	0.0647	0.0645	0.0615	0.0590

**Fig. 15:**
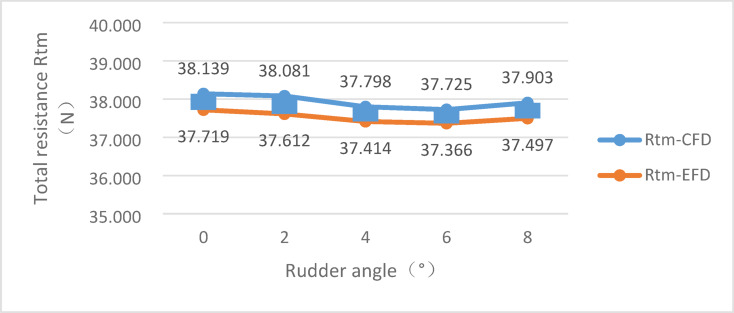
Comparison of the total resistance from CFD and EFD.

It can be obtained from the comparison in Fig. 15, the total resistance change shows a similar trend in both the CFD simulation and the EFD tests. In comparison with the total resistance of the bare hull, the total resistance increases after the rudder is installed. As the rudder angle increases, the rudder resistance gradually decreases. When the rudder angle gets 6 °, the rudder resistance ireaches smallest value. The lift of the rudder shows a downward trend, and gradually decreases with the increase of the rudder angle. Similarly, the lift is the smallest at the rudder angle 6°.

### Rudder angle impact on hull-rudder interaction

#### Axial wake field in front of the Rudder without propeller

**Fig. 16:**
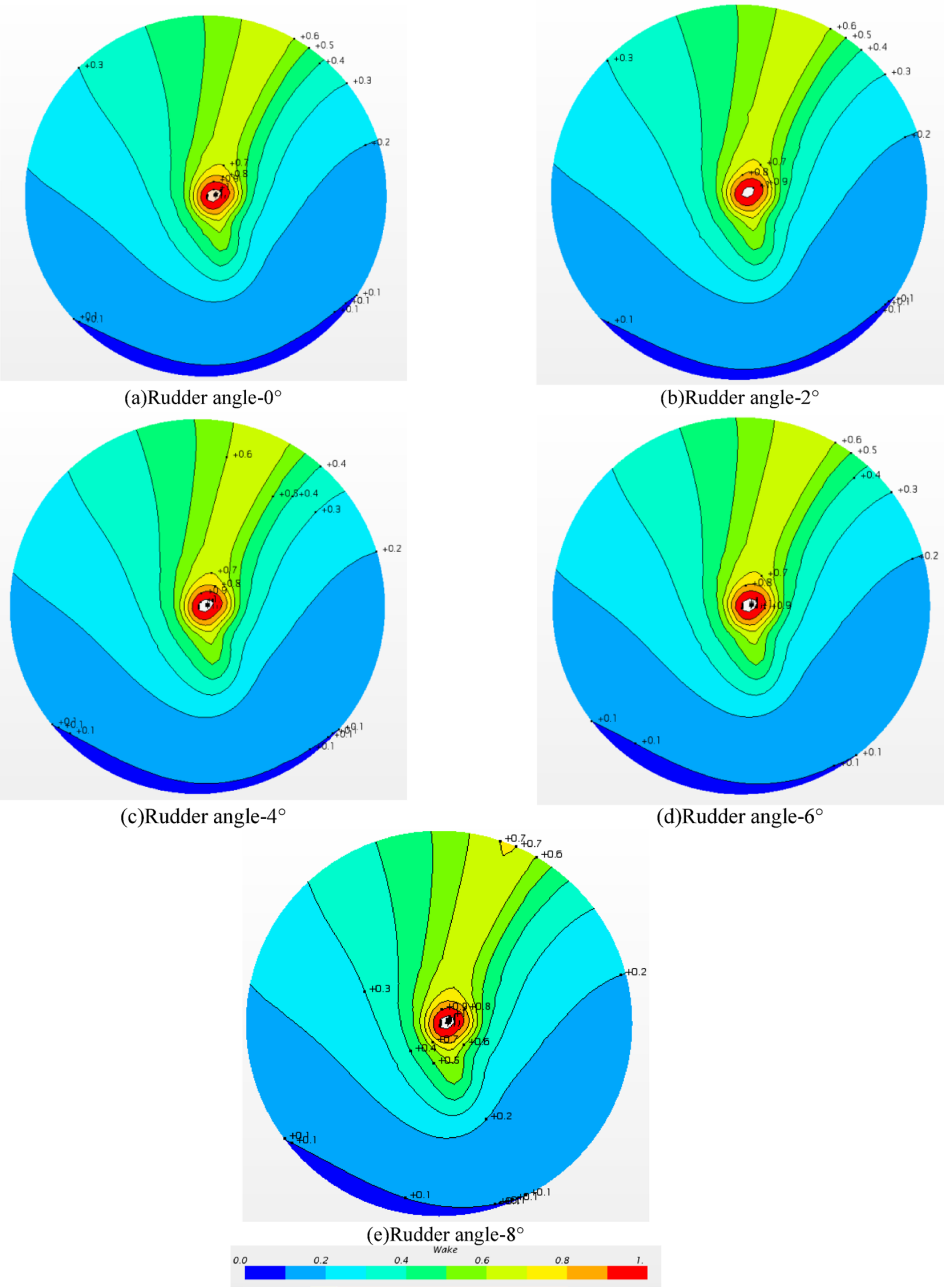
Axial wake field in front of the rudder without propeller.

The wake plane near the propeller position and in front of the rudder is shown in Fig. 16, in which the contour calculation formula of the wake field *w*_tm_ is shown in the Eq. (8). The larger the *w*_tm_ value, the smaller the axial velocity in this position.

8 $${w_{tm}}=1 - {V_a}/{V_m}$$

In Eq. (8), *V*_a_ is the axial velocity at this position, and *V*_m_ is the towing speed of the ship model, in this study, *V*_m_ = 1.534 m/s.

It can be seen that the high wake region is obliquely inclined to the mid-ship along the oblique stern in the YZ plane. Due to the influence of the stern, the flow field behind the stern forms a high wake area with the decrease of velocity, and the flow velocity gradually increases in the area extending outward along the stern and finally develops into a low wake area. According to the analysis in the above figures, it can be found that the wake in front of the rudder changes little at different rudder angles, which indicates that the change of rudder angle has little effect on the flow field in front of the rudder. Therefore, the change of the rudder angle mainly affects the flow field behind the rudder and the rudder force, it has little influence on the ship.

#### Velocity vector of Rudder profile at shaft height

**Fig. 17:**
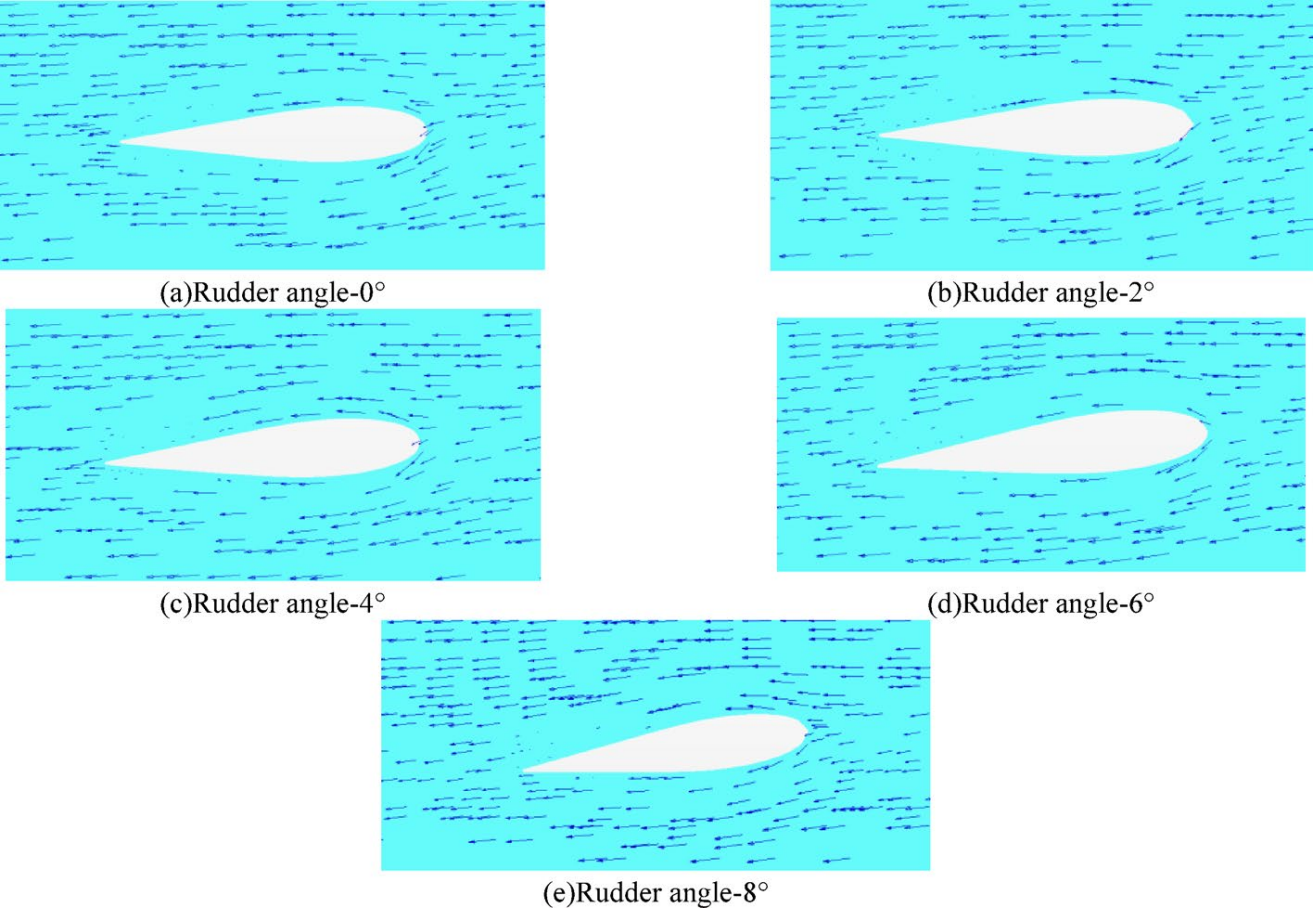
Velocity vetor rudder profile at shaft height.

Observing the port rudder from top to bottom and intercepting the velocity vector of the rudder section at the height of the shaft, the results can be seen in the following figures of Fig. 17.

As can be seen from the velocity vector above, the flow in front of the rudder and the inside of the rudder show a certain attack angle. With the increase of the rudder angle, the attack angle of the rudder gradually changes in the non-uniform wake field. The attack angle at different sections is different due to the difference of the flow field. When the rudder angle issmaller than 2 °, the outer side of the rudder leading edge faces the flow, and the inner velocity vector shows a divergent state. In the case of the rudder angle being 4° − 6°, the rudder leading edge near the shaft height gradually turns to the upstream state, and the convergence of the velocity vector on both sides of the rudder is enhanced. When the rudder angle ireaches 8°, the inner velocity vector of the rudder continues to converge and is closer to the rudder surface, but the outer velocity vector gradually diverges. In summary, the rudder working in the wake field behind the stern has different degrees of interaction with the flow field due to different rudder angles. With the rudder angle being 4° − 6°, the velocity vector around the rudder is closer to the rudder and is streamlined.

#### Dynamic pressure distribution on Rudder surface

**Fig. 18:**
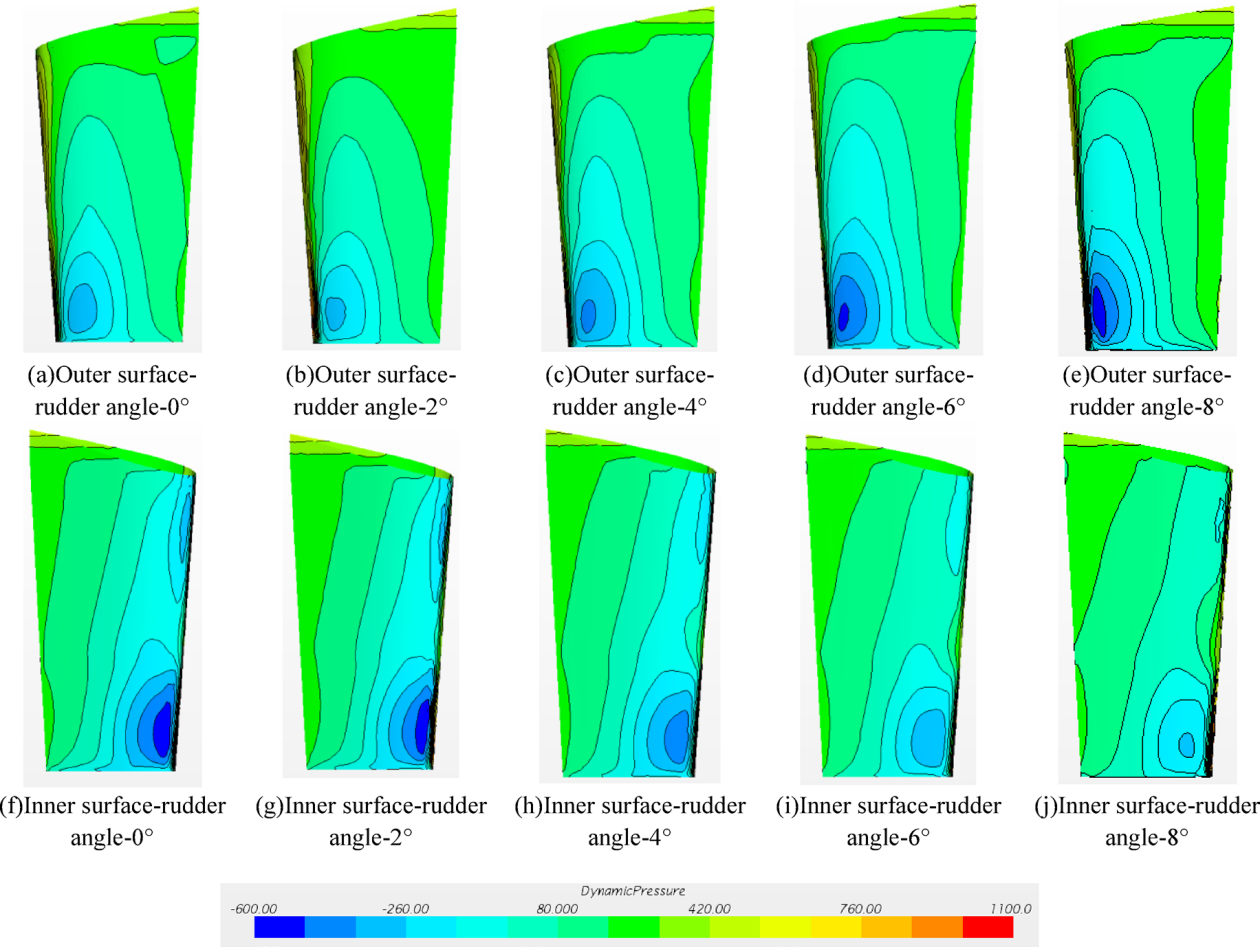
Dynamic pressure distribution on rudder surface.

As shown in Fig. 18, if the rudder behind the ship is in the non-uniform wake field, the pressure distribution at different positions of the rudder surface is greatly different due to the different rudder angles. Observing from the inside of the port rudder, there is an obvious high negative pressure area below the shaft height, and a small negative pressure area above the shaft height due to the shelter of the hull. As the leading edge of the rudder rotates inward, the high negative pressure area inside the rudder decreases. This is because when the rudder turns to the flow direction, the flow velocity decreases and the angle of attack changes, which makes the pressure tend to be uniform. The peak pressure distribution on the outer side of the rudder is mainly concentrated below the shaft height, and gradually increases with the increase of the rudder angle.

#### The influence of Rudder on flow field and total resistance

**Fig. 19:**
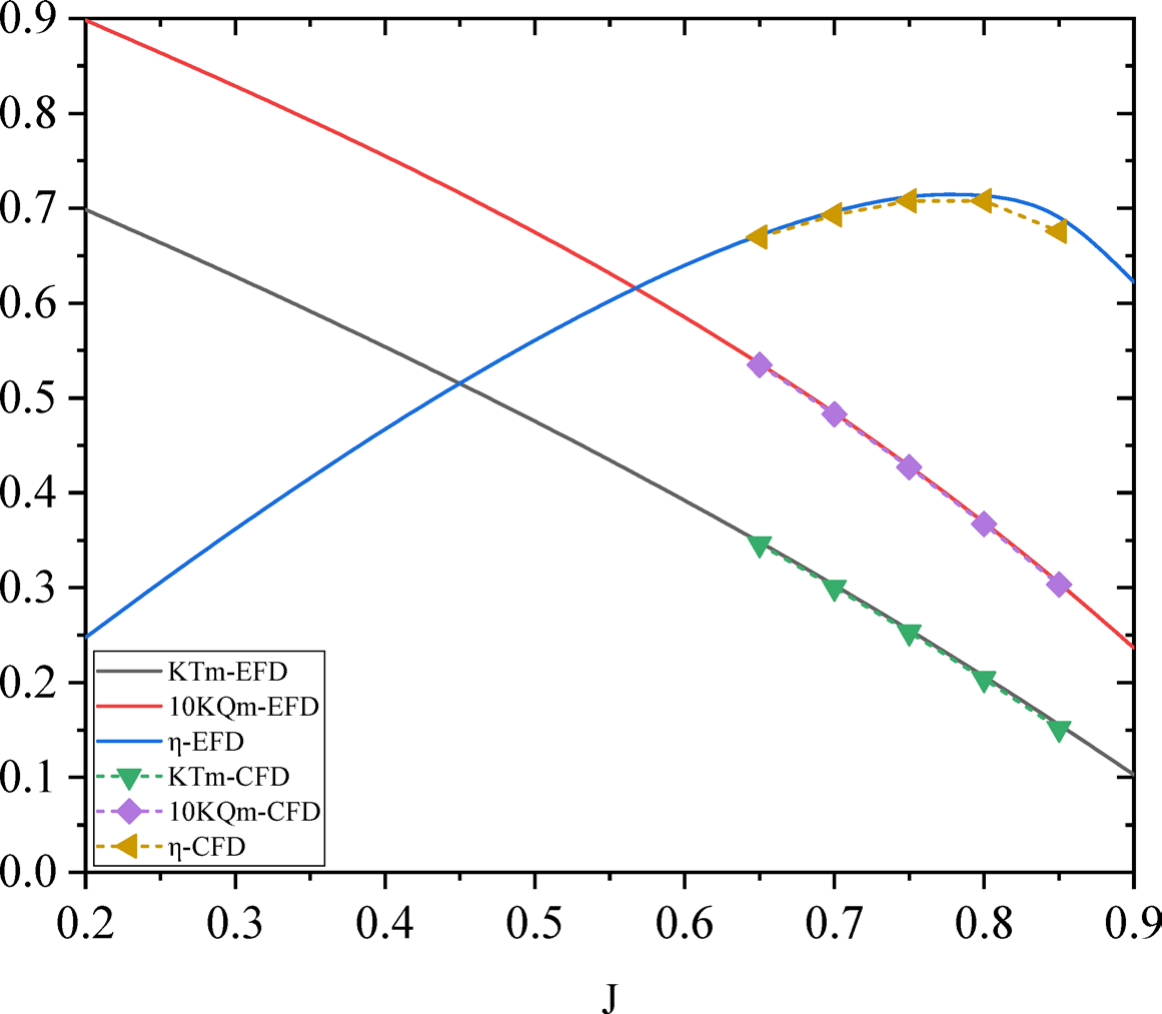
Propeller open water characteristics of CFD and EFD results.

In summary, the results of different rudder angles are well cross-verified. Because of the shielding effect of the stern, the velocity vector near the rudder has a certain angle with the longitudinal direction of the ship in the wake field of the twin-propeller ship, which acts on the rudder to form different attack angles. The appropriate angle of attack can effectively reduce the flow separation on the rudder surface, making the pressure distribution on the rudder surface more uniform and reducing the total resistance. Therefore, it can obviously reduce the total resistance by adjusting the symmetrical rudder angle for the twin-propeller/ twin-rudder ship (Fig. 19).

## Impact of Rudder angle on self-propulsion performance and hull-propeller-rudder intercation

The RBM method is used to numerically simulate the overall scheme of the hull-propeller-rudder interaction. The advantage is that it can simulate the mechanical interaction between the propeller and the hull-rudder with acceptable accuracy. The mesh near the stern is shown in Fig. 20, the refinement method is similar to the CFD simulation of resistance. The numerical simulation of self-propulsion is also verified by grid independence, with the final mesh number being about 3.5 million for half domain in port side.

### Propeller open water characteristics

The open water performance analysis of propellers were compared with numerical calculations and model tests. In the numerical simulations, the RBM method was used to calculate the thrust coefficient *K*_Tm_, torque coefficient *K*_Qo_ and open water efficiency *η*_*o*_ when the advance coefficient *J*_m_ = 0.65–0.85, where *J*_m_ =*V*_a_/(*nD*), *V*_a_ is the advanced speed, *n* is the propeller rotation speed, *D* is the propeller diameter. The open water performance obtained during the open water test of the propeller model in advance coefficient *J*_m_ =0.20–0.90. Finally, the results of numerical simulations and open water tests were compared, and the results were shown in the figure. In which, the results were the total characteristics of the twin propellers. CFD results of the thrust coefficient, torque coefficient and open water efficiency were slightly lower than the EFD results by about 1%, the CFD calculation results were within the acceptable accuracy, as shown in Fig. 19.

According to the force applied on the ship model, the thrust, torque and overall force of the propeller at different speeds are obtained using the external force self-propulsion method at the design speed. At the target towing speed, three different rotation speeds were applied to the propeller to obtain the total force of ship, propeller thrust and torque at this towing speed. The self-propulsion data processing method in Reference^15^ is used to interpolate the system force at different speeds with the external force *F*_D_, and the corresponding rotation speed, thrust and torque of *F*_D_ are obtained.

### Self-propulsion analysis method

**Fig. 20:**
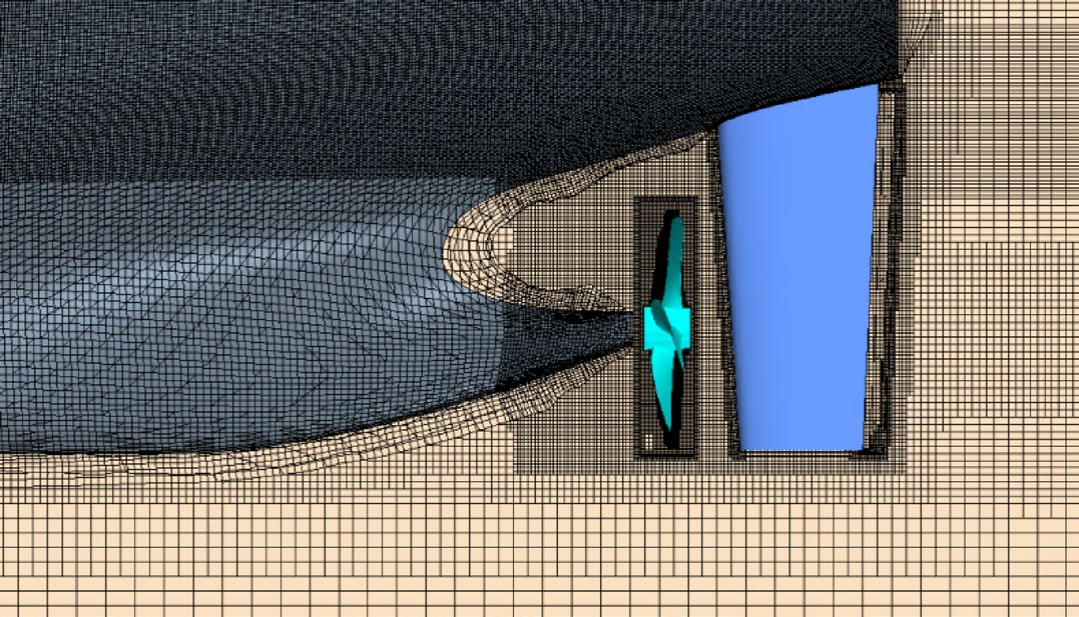
Mesh grids of ship aft-bofy with rudder and propeller.

### Self-propulsion model tests

The self-propulsion test is adopted to verify the propulsion performance of the hull-appendages-propeller structure. The formulae of the self-propulsion point *F*_D_, the total power *P*_m_, the propulsion efficiency *η*_m_, the thrust reduction *t*, the thtust coefficient *K*_Tm_, the torque coefficient of propeller open water *K*_qo_, the open water efficiency *η*_o_, torque coefficient of propeller behind the stern *K*_Qm_, relative rotation efficiency *η*_R_ and hull efficiency *η*_H_ in the model scale are given as follows.

9 $$F_{\user2{D}} = 0.5\rho _{m} S_{m} V_{m} ^{2} (C_{{{\text{fm}}}} - C_{{{\text{fs}}}} - \Delta C_{F} )$$

10 $${P_m}=2\pi {n_m}{Q_m}$$

11 $${\eta _m}=\frac{{({R_{tm}} - {F_D}){V_m}}}{{2\pi {n_m}{Q_m}}}$$

12 $$t=1 - \left( {{R_{tm}} - {F_D}} \right)/{T_m}$$

13 $${K_{Tm}}=\frac{{{T_m}}}{{\rho n_{m}^{2}{D^4}}}$$

14 $${K_{Qm}}=\frac{{{Q_m}}}{{\rho n_{m}^{2}{D^5}}}$$

15 $${K_{Qo}}=\frac{{{Q_o}}}{{\rho n_{m}^{2}{D^5}}}$$

16 $$\eta o=\frac{{J{K_{Tm}}}}{{2\pi {K_{Qo}}}}$$

17 $${\eta _R}=\frac{{{K_{Qo}}}}{{{K_{Qm}}}}$$

18 $${\eta _H}=\frac{{1 - t}}{{1 - {w_{tm}}}}$$

Where *n*_m_, *Q*_m_ and *T*_m_ are rotational speed, torque and thrust, respectively. *F*_D_ is the external force at the model scale. *ρ*_m_, *S*_m_, *V*_m_ and *C*_fm_ are the fresh water density, the wetted area of the ship model, the speed of the ship model and the friction resistance coefficient of the ship model, respectively. *C*_fs_ is the friction resistance coefficient of the real ship, and $\:\varDelta\:{C}_{F}$ is the friction resistance coefficient allowance predicted for the real ship. *ρ* is the density of water density.

### Results of self-propulsion with CFD and EFD method

**Fig. 21:**
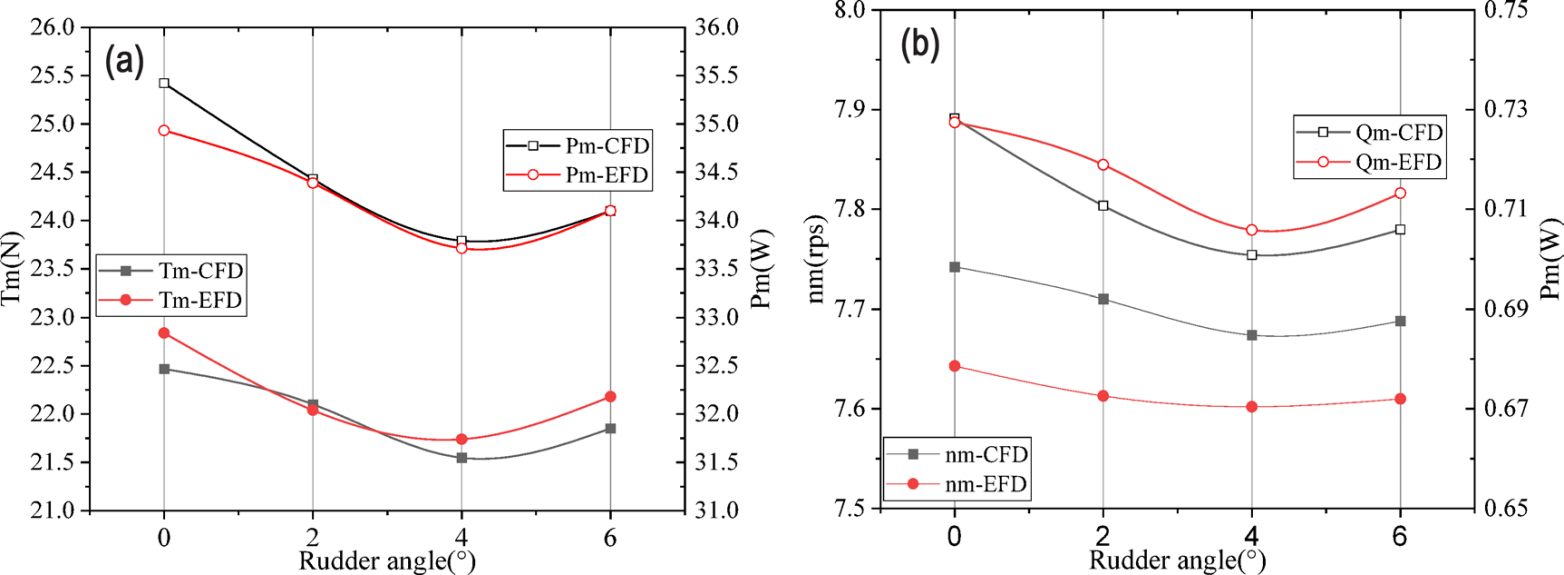
Self-propulsion results of CFD and EFD. (**a**) T_m_ and P_m_ for CFD and EFD results (**b**) n_m_ and Q_m_ for CFD and EFD results.

In this section, the self-propulsion results at different rudder angles are presented and analyzed in Table 4; Fig. 21. In the case of the rudder angle being 6°, the total power *P*_m_ starts to rise, so the self-propulsion simulation is perfomred until the rudder angle of 6°. In order to verify the accuracy of the numerical results, the experiments were carried out in the deep water towing tank of Shanghai Ship and Shipping Research Institute Co., Ltd.

**Table 4:** Self-propulsion results of CFD and EFD at model scale.

Data	Rudder angle			
	0°	2°	4°	6°
n_m_-CFD(rps)	7.742	7.710	7.674	7.688
n_m_-EFD(rps)	7.643	7.613	7.602	7.610
Error of n_m_	1.295%	1.274%	0.947%	1.025%
T_m_-CFD(N)	22.468	22.101	21.547	21.852
T_m_-EFD(N)	22.84	22.04	21.74	22.18
Error of T_m_	-1.63%	0.28%	-0.89%	-1.48%
Q_m_-CFD(N*m)	0.7282	0.7107	0.7008	0.7059
Q_m_-EFD(N*m)	0.7274	0.7189	0.7058	0.7132
Error of Q_m_	0.11%	-1.14%	-0.71%	-1.02%
P_m_-CFD(W)	35.423	34.429	33.791	34.099
P_m_-EFD(W)	34.931	34.388	33.712	34.102
Error of P_m_	1.407%	0.119%	0.232%	-0.009%

Through the analysis of the above table, the CFD results show the same trend as that from the EFD approach. The total power Pm continues to decrease when the rudder angle is below 4°, and the power increases when the rudder angle reaches 6°.Therefore, the optimal rudder angle is 4° for the hull-propeller-rudder combination. It is different from that of resistance arrangement, which is 6°. From the comparison between CFD and EFD results, the speed is about 1% higher, the thrust is a 1% lower and the torque is 1% lower. The total power Pm at the model scale has a small error when the rudder angle is 2° -6°. In general, the CFD and EFD results show the same trend, and the error is less than 2%.

The self-propulsion factors of CFD and EFD results are shown in Tables 5 and 6,

**Table 5:** Self-propulsion factors of CFD simulations.

Rudder angle(°)	0	2	4	6
*K*_Tm_	0.2494	0.2473	0.2434	0.2461
10*K*_Qm_	0.4103	0.4038	0.4019	0.4046
*J*_m_	0.7541	0.7562	0.7601	0.7574
10*K*_Qo_	0.4221	0.4194	0.4154	0.4185
*η*_o_	0.7091	0.7098	0.7089	0.7089
*η*_R_	1.0288	1.0388	1.0337	1.0345
*w*_tm_	0.2502	0.2513	0.2509	0.2512
*η*_H_	1.1152	1.1318	1.1428	1.1193

**Table 6:** Self-propulsion factors of EFD results.

Rudder angle(°)	0	2	4	6
*K*_Tm_	0.2601	0.2530	0.2503	0.2548
10*K*_Qm_	0.4205	0.4189	0.4125	0.4159
*J*_m_	0.7424	0.7498	0.7528	0.7481
10*K*_Qo_	0.4316	0.4232	0.4197	0.4252
*η*_o_	0.7122	0.7135	0.7144	0.7135
*η*_R_	1.0263	1.0102	1.0176	1.0224
*w*_tm_	0.2732	0.2689	0.2670	0.2708
*η*_H_	1.1064	1.1332	1.1334	1.1138

**Fig. 22:**
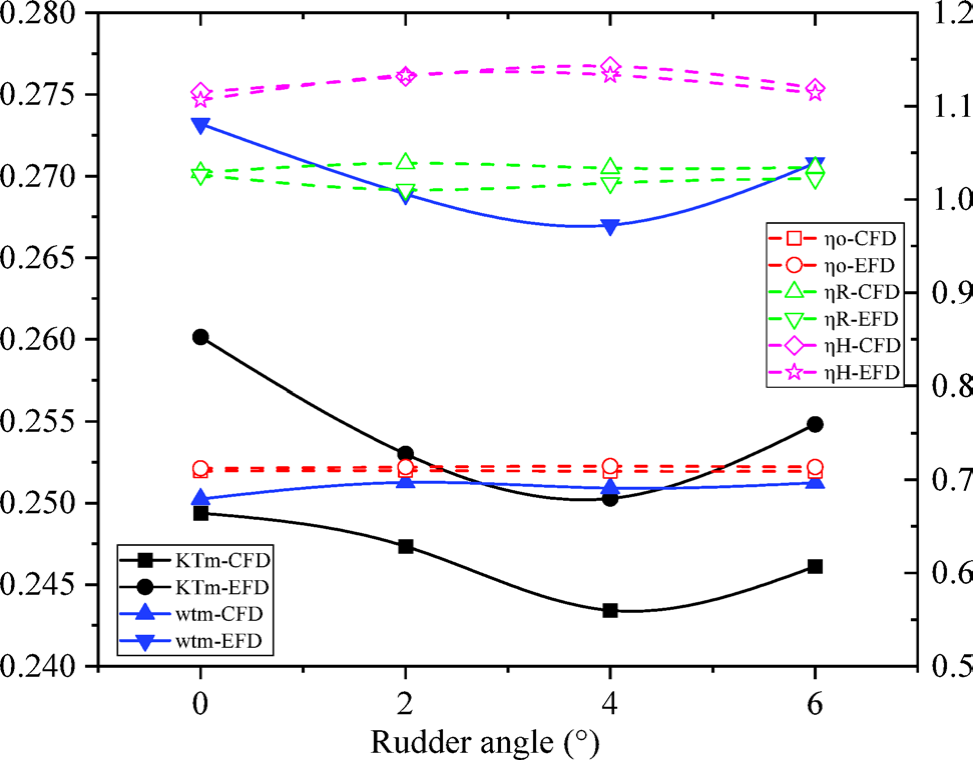
Self-propulsion factors of CFD and EFD results with rudder angles.

Through the comparison of Tables 5 and 6; Fig. 22, the results of CFD and EFD are similar, but the results of self-propulsion factor are different due to the difference of resistance, open water and self-propulsion calculation results. Among them, when the rudder angle is 4 °, the hull efficiency *η*_H_ is the highest. Open water efficiency and relative rotation efficiency have the same trend. For the wake fraction results of numerical simulation, the wake fraction *w*_tm_ at different rudder angles is approximate. For the EFD wake fraction results, when the rudder angle is 4 °, the wake fraction is the smallest.

### Analysis of hull-propeller-rudder interaction

In order to compare the interaction of hull-propeller-rudder, in the flow field analysis, the flow field results at the propeller rotational speed of 7.8rps were compared.

#### Axial wake field in front of the Rudder with propeller

**Fig. 23:**
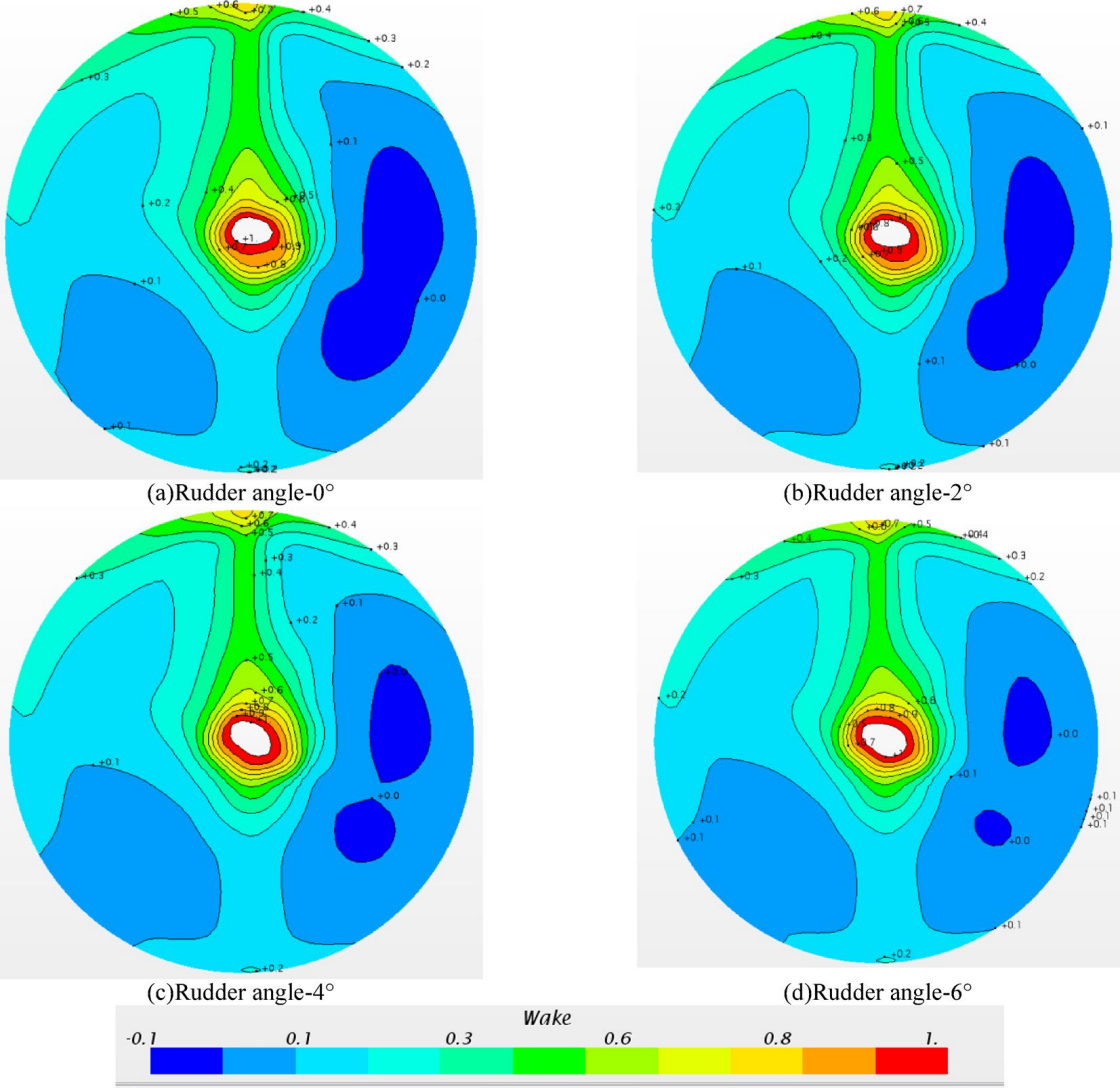
Axial wake field in front of the rudder with propeller.

The wake distribution in front of the rudder and behind the propeller is presented in Fig. 23. Compared with Fig. 16, due to the rotation of the propeller. The wake distribution of the hull-propeller-rudder and the hull-rudder changed obviously. The high wake area changed from the upper part to the middle of the ship to the vertical upper hull. When the propeller rotates, the surrounding flow field is driven to rotate, and the wake value *w*_tm_ will gradually decrease along the axial direction of the propeller, as shown in the lower half of the plane in the figures. However, due to the influence of the hull, the fluid around the propeller rotates and encounters the hull, and the fluid velocity gradually decreases, which makes the wake in the vertical direction large, and then the high wake area changes along the propeller shaft and vertical hull direction.

Compared with the a ) -d ) diagram in Fig. 23, the wake distribution is approximate, and only the negative wake regions on the right side are slightly different. The wake values of these negative wake regions are between − 0.1 and 0. When the wake value is less than 0, it means that there is backflow here, and the obvious backflow will adversely affect the propulsion performance of the propeller. The backflow of Fig. a ) and Fig. b ) is relatively obvious, and the backflow of Fig. c ) and Fig.d ) is relatively small. In general, the change of rudder angle has little effect on the wake distribution in front of the rudder. However, due to the small difference in the negative wake area caused by different rudder angles, the propulsion performance of the propeller will change.

#### Dynamic pressure distribution on propeller surface

**Fig. 24:**
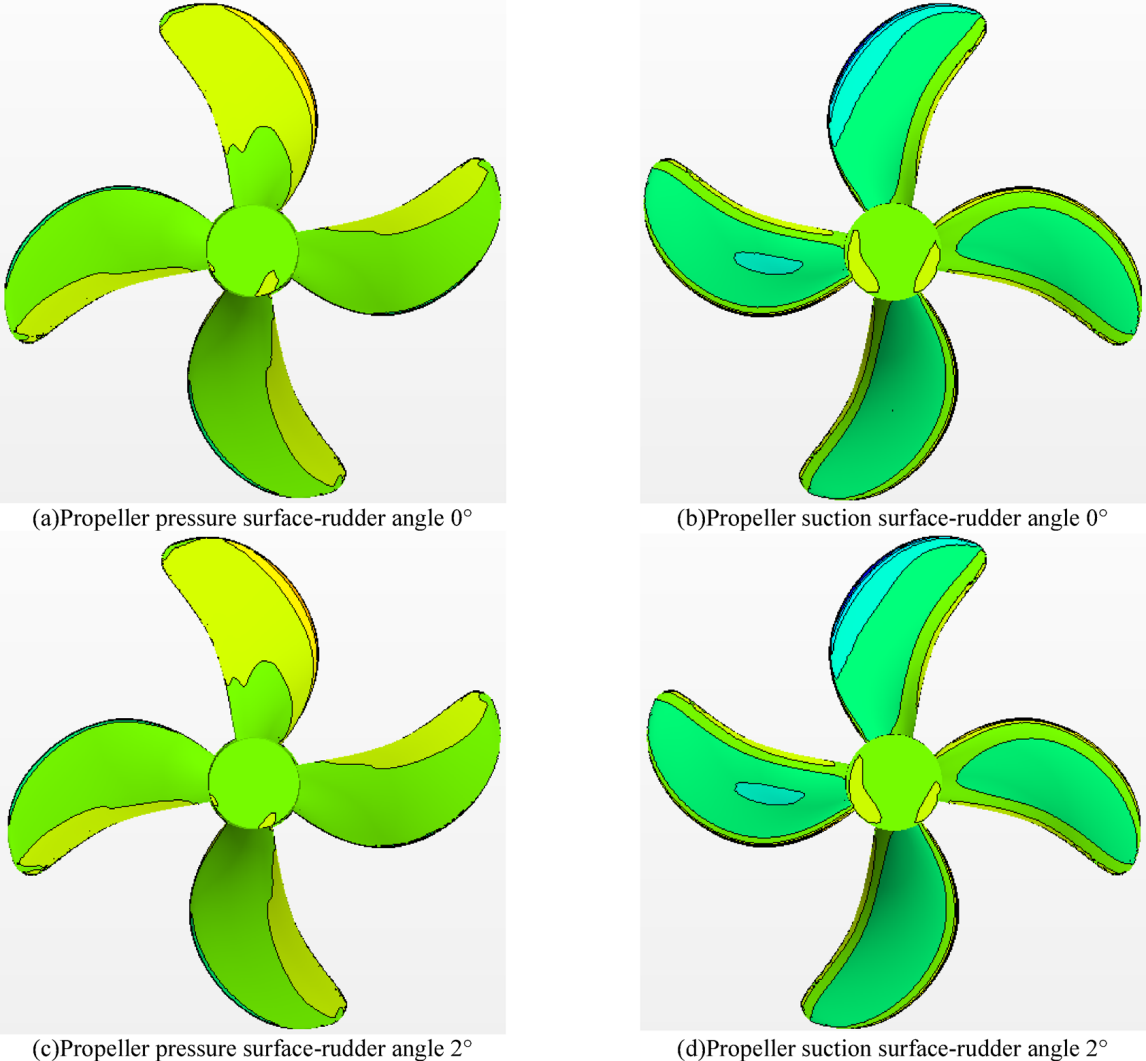

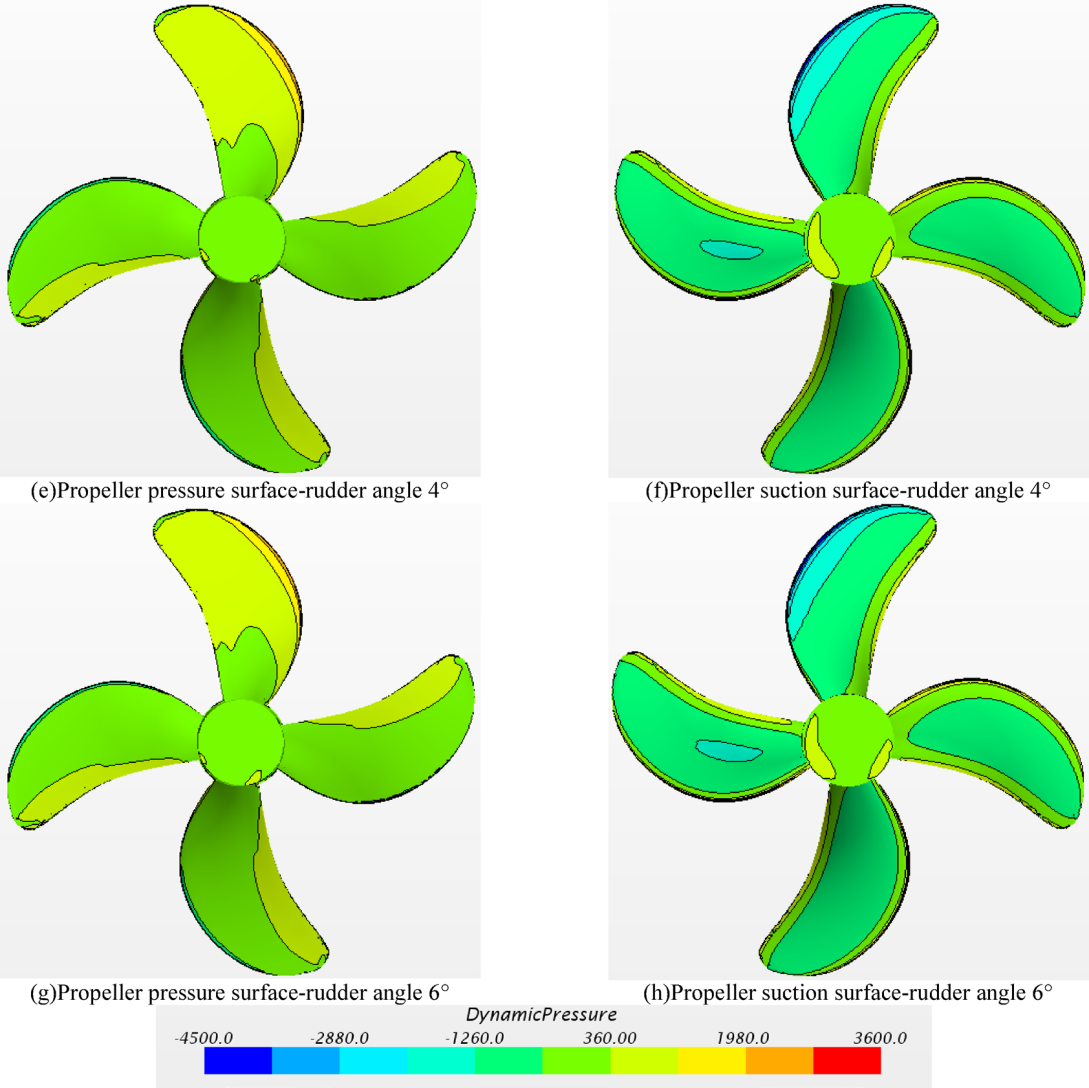
Dynamic pressure distribution on the propeller surface at different rudder angles.

**Fig. 25:**
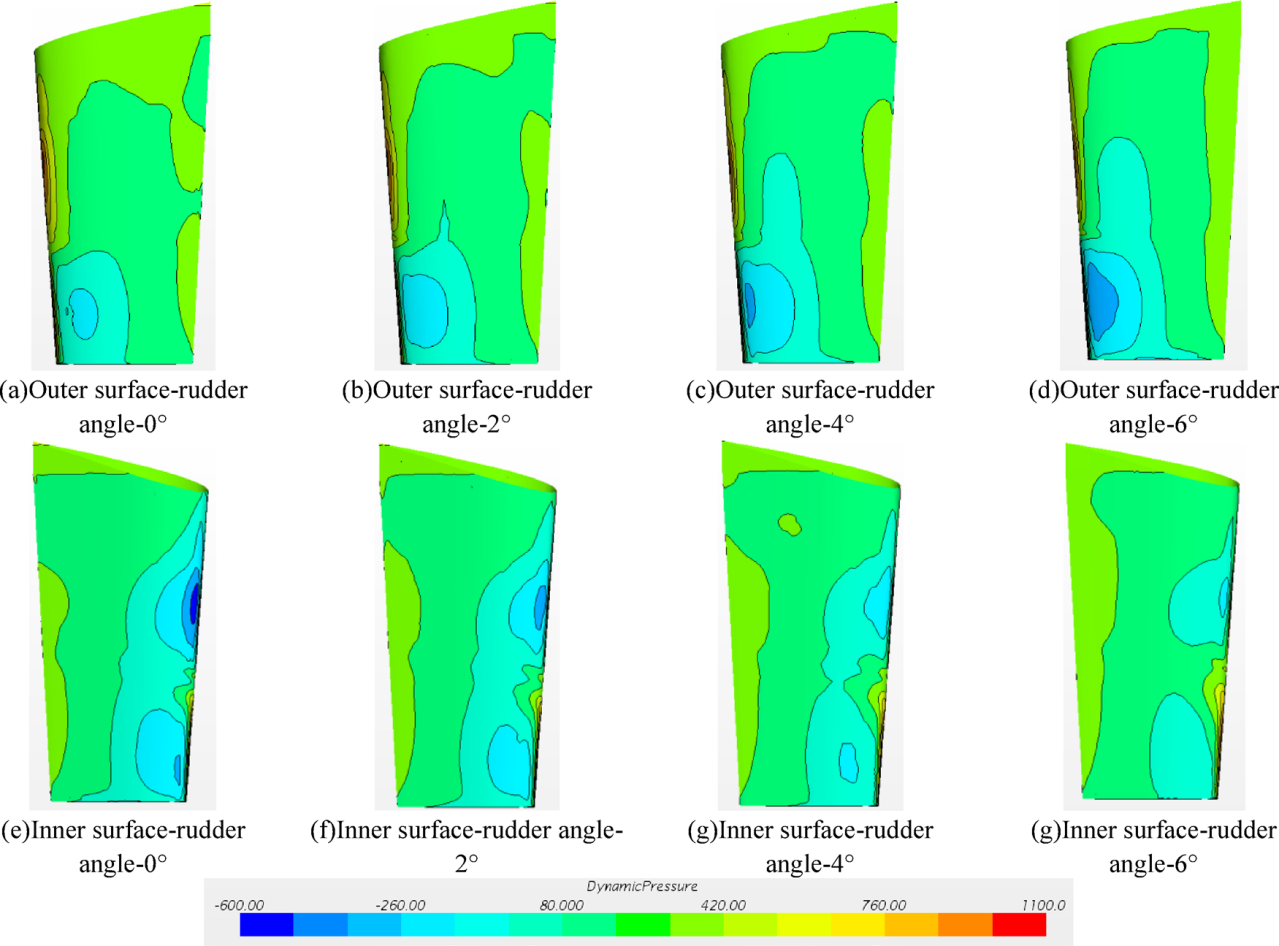
Dynamic pressure distribution on rudder surface (propeller installed).

When the rudder angle is 0° -4°, the surface dynamic pressure distribution contour map of the pressure surface and the suction surface of the propeller in the rotating state is shown in Fig. 24. For the propeller, the performance of the propeller is mainly affected by three aspects : (1) the wake flow in front of propeller, (2) the hull upper the propeller, and (3) the rear rudder behind propeller. According to the figures of Fig. 25a–h), the dynamic pressure distribution of the propeller pressure surface and the suction surface under different rudder angles is similar, which means that at this towing speed, when the propeller rotates at the same speed, the change of the rudder angle has little effect on the dynamic pressure distribution of the propeller.

#### Dynamic pressure on the rudder surface

As shown in Fig. 25, the difference between the self-propulsion and the resistance result is clear. If the rudder angle is smaller than 2°, the negative pressure peak appears above and below the inner side of the rudder. The absolute value of the negative pressure above the inner side of the rudder is greater thanthat below the inner side of the rudder. The negative pressure area below the outer side of the rudder is lower than that from the resistance results. When the rudder angle increases to 4°, the dynamic pressure distribution inside and outside the rudder tends to be uniform and there is no obvious peak. In the case of the rudder angle 6°, the negative pressure area under the outer side of the rudder starts to increase obviously, and the inner pressure distribution is similar to the result withthe rudder angle 4°. This mainly results from the rotation of the fluid driven by the internal rotation of the propeller, where the flow velocity above the rudder increases and the flow velocity below the rudder decreases relatively and the negative pressure peak gradually switches. Different rudder angles may cause differences in the dynamic pressure distribution on the rudder surface, resulting in changes in the resistance and rectification of the rudder. This is also the reason for the differences in propulsion power *P*_m_, rotational speed *n*_m_, thrust *T*_m_ and torque *Q*_m_.

#### Velocity vetor Rudder profile at shaft height

**Fig. 26:**
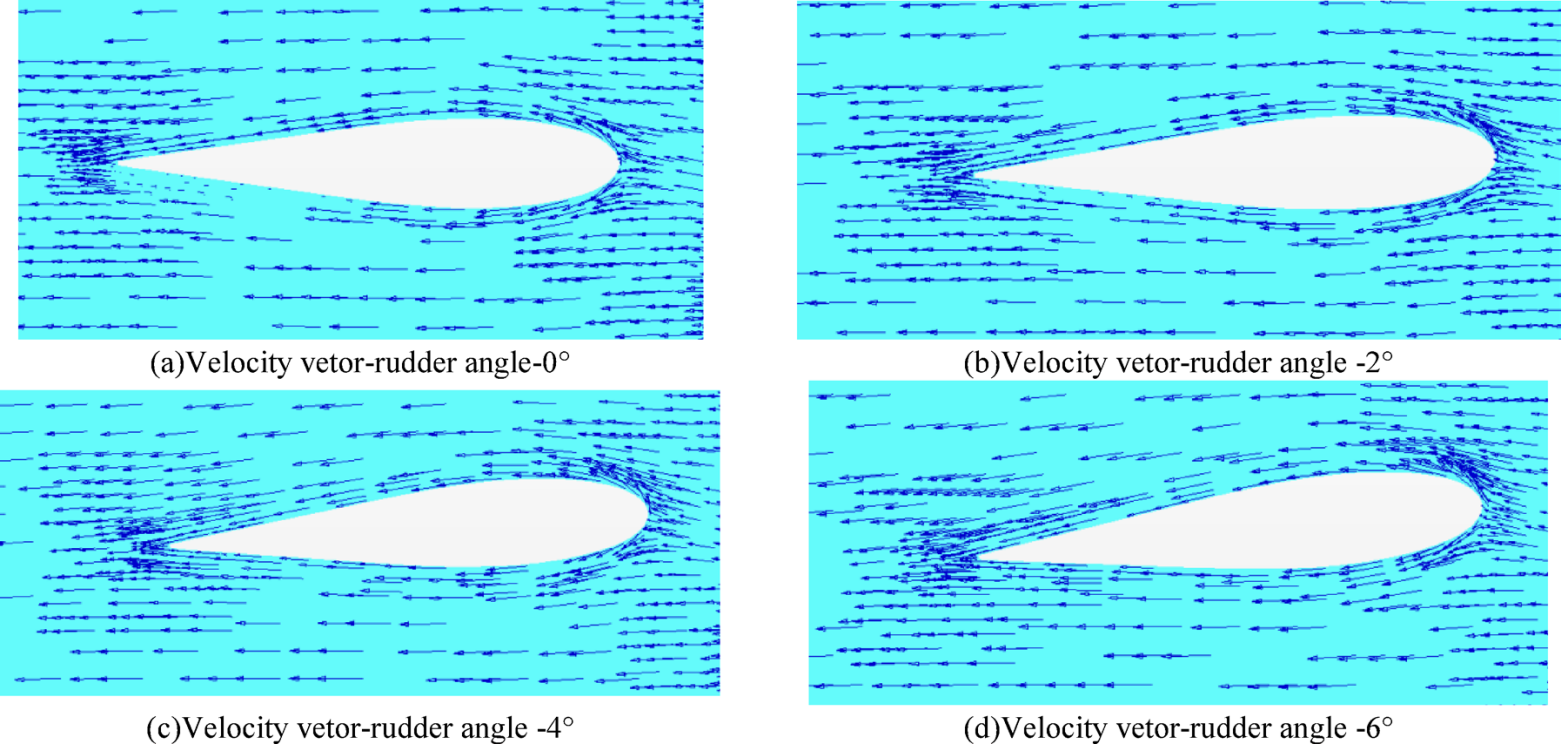
Velocity vetor rudder profile at shaft height(propeller installed).

The section velocity vector diagram of different rudder angles near the engine shaft height is shown in Fig. 26 as follows.

Compared with the resistance simulation, the velocity vector of the self-propulsion result is more complex. With the increase of the rudder angle, the influence of the velocity vector near the rudder leading edge and the direction of the inflow increases. The velocity vector inside the rudder gradually increases. Especially when the rudder angle is smaller than 2°, the velocity vector behind the inner side of the rudder is relatively small. In the case of the rudder angle being 4° -6°, the velocity vector gets more uniform and closer to the rudder surface.

#### Streamline and vortex of hull-propeller-rudder interaction

**Fig. 27:**
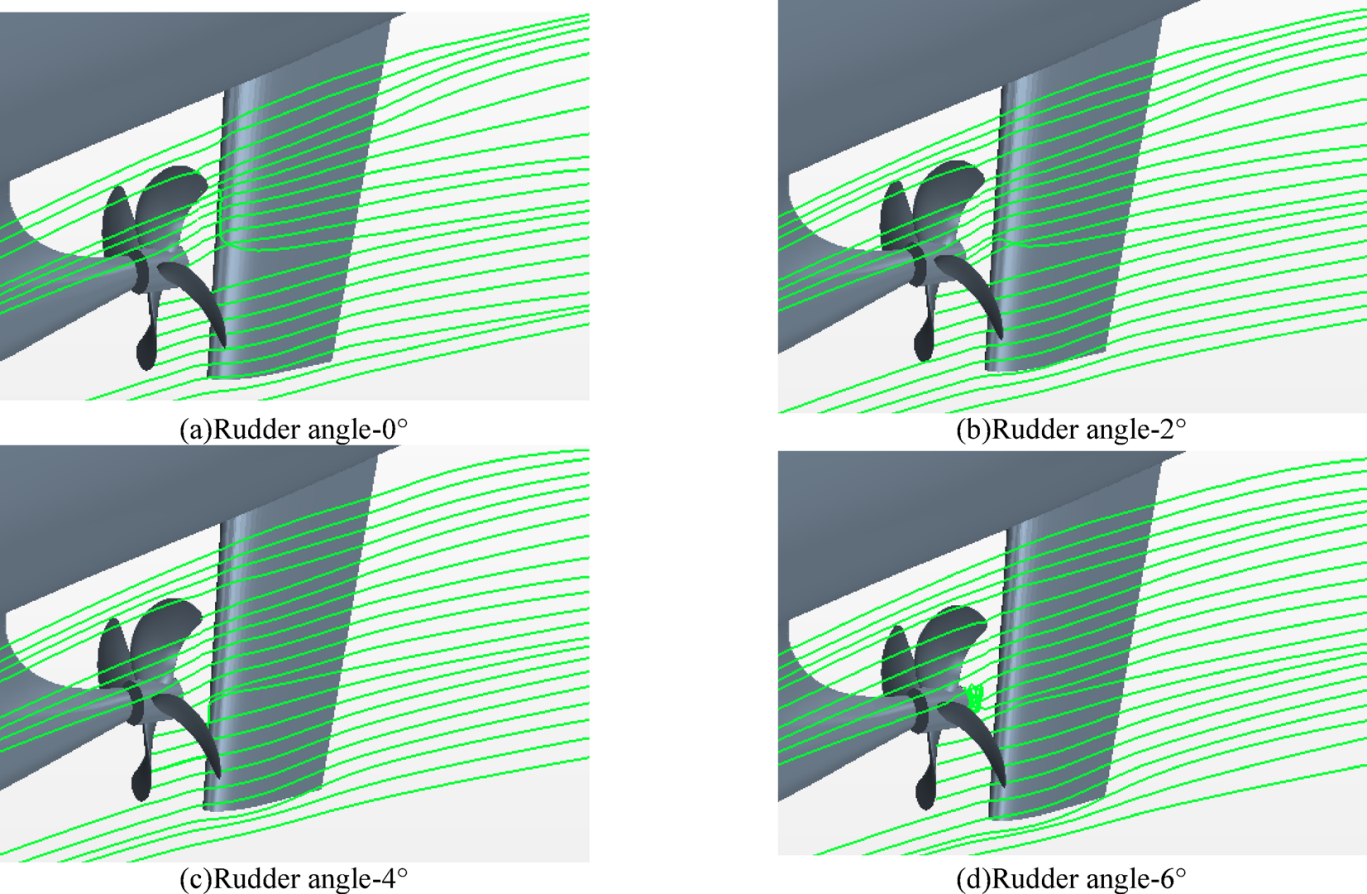
Streamlines near hull-propeller-rudder at different rudder angles.

In order to further analyze the mechanism of hull-propeller-rudder interaction, the flow field information such as streamline and wake vortex near the hull-propeller-rudder is analyzed in Fig. 27. It can be clearly seen that when the rudder angle increases from 0 ° to 4 °, the streamline around the propeller are similar, and the streamline near the rudder changes from ' non-uniform ' to ' uniform ‘. However, when the rudder angle is 6 °, an obvious vortex streamline appears near the propeller cap, indicating that there are more adverse changes in the flow field.

**Fig. 28:**
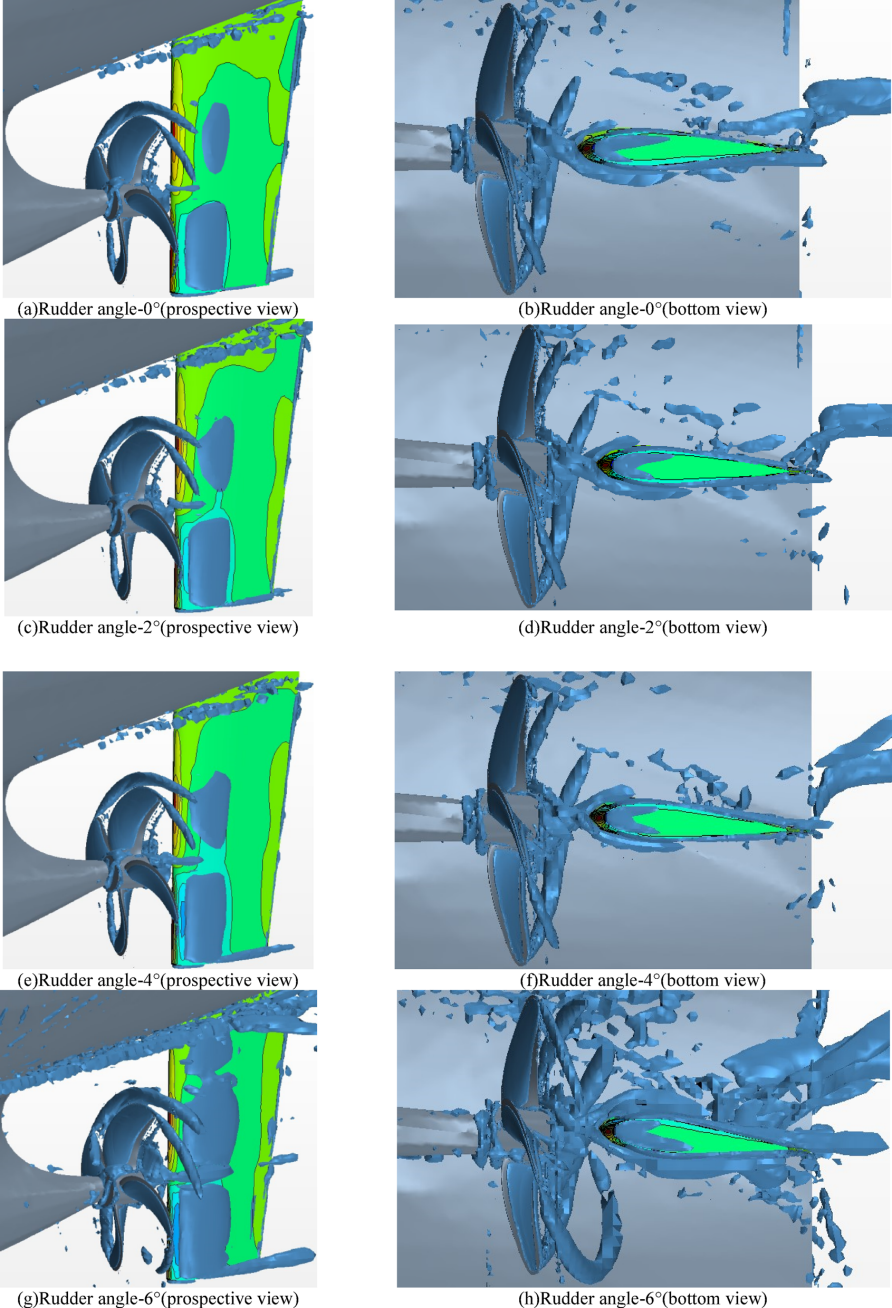
Wake vortex of hull-propeller-rudder at different rudder angles ( left : prospective view. Right : Bottom view ). Source: Figure 28 captured by the authors from star-ccm+.

Figure 28 shows the evolution of the wake vortex field when the rudder angle changes from 0° to 6°. The selection of the wake vortex is based on the Q criterion, Q = 100 / S^2^. The left side is the prospective view, and the right side is the bottom view. According to Fig. 28a ) and b ) when the rudder angle is 0 °, the vorticity on the outer side of the rudder is small, while the vorticity on the inner side is larger than that on the outer side. According to figures c ) to f ), when the rudder angles are 2 ° and 4 °, the vorticity on the outer side of the rudder increases slightly, while the vorticity on the inner side decreases significantly. In general, when the rudder angle increases from 0 ° to 4 °, the vorticity inside the rudder gradually decreases, the vorticity outside the rudder changes slightly, and the vorticity around the hull is similar. Figure g ) and h ) show the vorticity field when the rudder angle is 6 °. It can be seen that the wake vortex around the rudder increases significantly and the shape is chaotic. The wake vortex generated by the rotating propeller increases, and the wake vortex at the stern of the hull also increases significantly. Therefore, when the rudder angle is 6 °, the rudder has an adverse effect on both the propeller and the hull, resulting in a decrease in ship performance, which is also consistent with the results described above.

## Conclusions

The ship resistance and self-propulsion performance study and hull-propeller-rudder interaction under different rudder angles were carried out for a twin-rudder ship. CFD and EFD method were used to analyze the resistance with the rudder angle between 0° and 8° and the self-propulsion performance for the rudder angle between 0° -6°. Resistance and self-propulsion factors, pressure distribution on the rudder surface, wake field, velocity vector and vorticity results from CFD and EFD have been compared. The following conclusions are obtained:

1. Since the rudder is symmetrical arranged on the port and starboard, the rudder angle has a significant contribution to the resistance of the ship. In the non-uniform wake field behind the ship, the total resistance is the smallest for the rudder angle 6°. The propeller rotation drives the wake field to rotate, making the flow field more complex, resulting in changes of resistance flow field and self-propulsion flow field. When the rudder angle is 4°, the delivered power of self-propulsion is smallest, obtaining energy reduction.
2. At different rudder angles, the rudder has little impact on the propeller performance, but the rudder angle can change the flow field around the rudder, resulting in different hull-propeller-rudder interaction. Therefore, the ship performance and hull-propeller-rudder interaction are different at different angle, and the best angle is different for resistance and self-propulsion conditions.
3. Compared with the EFD results, the accuracy of the CFD simulation results is within the acceptable range, showing the same change trend in analyzing resistance, open water and self-propulsion of ship with different rudder angles, proving that the RANS method is a effective tool in simulating ship performance.
4. Appropriate rudder angle can effectively improve the dynamic pressure distribution on the rudder surface, thereby reducing the resistance and providing a choice for reducing ship ‘s delivered power.

## Data Availability

Data is provided within the manuscript files. If any further query or raw data are required, please contact ZL(vzhangli@hrbeu.edu.cn).
